# Gut microbiota alteration after cholecystectomy contributes to post-cholecystectomy diarrhea via bile acids stimulating colonic serotonin

**DOI:** 10.1080/19490976.2023.2168101

**Published:** 2023-02-02

**Authors:** Yayun Xu, Jianfa Wang, Xubo Wu, Hui Jing, Shilong Zhang, Zhiqiu Hu, Longhua Rao, Qimeng Chang, Lishun Wang, Ziping Zhang

**Affiliations:** a Department of Hepatopancreatobiliary Surgery, Minhang Hospital, Fudan University, Shanghai, P.R. China; b Institute of Fudan-Minhang Academic Health System, Minhang Hospital, Fudan University, Shanghai, P.R. China; c Department of Medical Oncology, Zhongshan Hospital, Fudan University, Shanghai, P.R, China; d Center for Traditional Chinese Medicine and Gut Microbiota, Minhang Hospital, Fudan University, Shanghai, China

**Keywords:** PCD, gut microbiota, gastrointestinal motility, 5-HT, BAs

## Abstract

Post-cholecystectomy diarrhea (PCD) is highly prevalent among outpatients with cholecystectomy, and gut microbiota alteration is correlated with it. However, how and to what extent changed fecal bacteria contributes to diarrhea are still unrevealed. Humanized gut microbiome mice model by fecal microbiota transplantation was established to explore the diarrhea-inducible effects of gut microbiota. The role of microbial bile acids (BAs) metabolites was identified by UPLC/MS and the underlying mechanisms were investigated with selective inhibitors and antagonists as probes. These mice transplanted with fecal microbiome of PCD patients (PCD mice) exhibited significantly enhanced gastrointestinal motility and elevated fecal water content, compared with these mice with fecal microbiome of NonPCD patients and HC. In analyzing gut microbiota, tryptophan metabolism was enriched in PCD microbiome. In addition, overabundant serotonin in serum and colon, along with elevated biosynthesis gene and reduced reuptake gene, and highly expressed 5-HT receptors (5-HTRs) in colon of PCD mice were found, but not in small intestine. Notably, diarrheal phenotypes in PCD mice were depleted by tryptophan hydroxylase 1 inhibitor (LX1606) and 5-HTRs selective antagonists (alosetron and GR113808). Furthermore, increased microbial secondary BAs metabolites of DCA, HDCA and LCA were revealed in feces of PCD mice and they were found responsible for stimulating 5-HT level *in vitro* and *in vivo*. Intriguingly, blocking BAs-conjugated TGR5/TRPA1 signaling pathway could significantly alleviate PCD. In conclusion, altered gut microbiota after cholecystectomy contributes to PCD by promoting secondary BAs in colon, which stimulates colonic 5-HT and increases colon motility.

## 1. Introduction

Post-cholecystectomy diarrhea (PCD) is a very common complication of gallbladder removal and its incidence reaches as high as 57.2%.^1,2^ The growing prevalence of cholecystolithiasis^3^ and preferable option to cholecystectomy in gallstone patients make PCD affect a large population globally. The chief complaint of PCD is disordered bowel habit, including increased defecation frequency, urgency, and loose stools, which affects postoperative quality of life deeply.^4,5^ Unfortunately, profound understanding on the pathogenesis of postoperative diarrhea is still unclear and effective therapeutical regimen for PCD is deficient.

Gut microbiota are being recognized as vital masters in maintaining host health, especially normal gastrointestinal functions, and fecal microbiome alterations are being accepted as essential etiological causes for numerous gastroenteric disorders, such as inflammatory bowel disease (IBD) and irritable bowel syndrome (IBS).^6,7^ Altered fecal bacteria in PCD patients has been reported by us and other authors^8,9^ and the influences of cholecystectomy on fecal bacteria were revealed by Yoon WJ et al.^10^ Nevertheless, whether altered gut microbiota in PCD patients is merely a consequence of diarrhea *per se* or a contributing factor for PCD remains unsettled, besides, how and to what extent intestinal flora changes are responsible for PCD are to be unraveled.

The microbiota-host crosstalk in maintaining normal physiology is defined precisely by microbial metabolites, among which tryptophan metabolites is receiving intensive attentions.^11–13^ Serotonin functions critically in maintaining normal intestinal peristalsis, and its biosynthesis is reported to be controlled by fecal bacteria, for example reduced serotonin level in fecal bacteria depleted mice and germ-free mice.^14,15^ However, the roles of microbial metabolites and serotonin in PCD remain unknown.

Herein, we found the impact of altered gut microbiota in PCD patients to diarrhea and we revealed that the bacterial secondary bile acids induced high serotonin in colon was the underlying mechanisms.

## 2. Results

### 2.1. Gut microbiota of post-cholecystectomy diarrhea (PCD) patients contributed to diarrhea in humanized microbiome mice.

**Figure 1.**
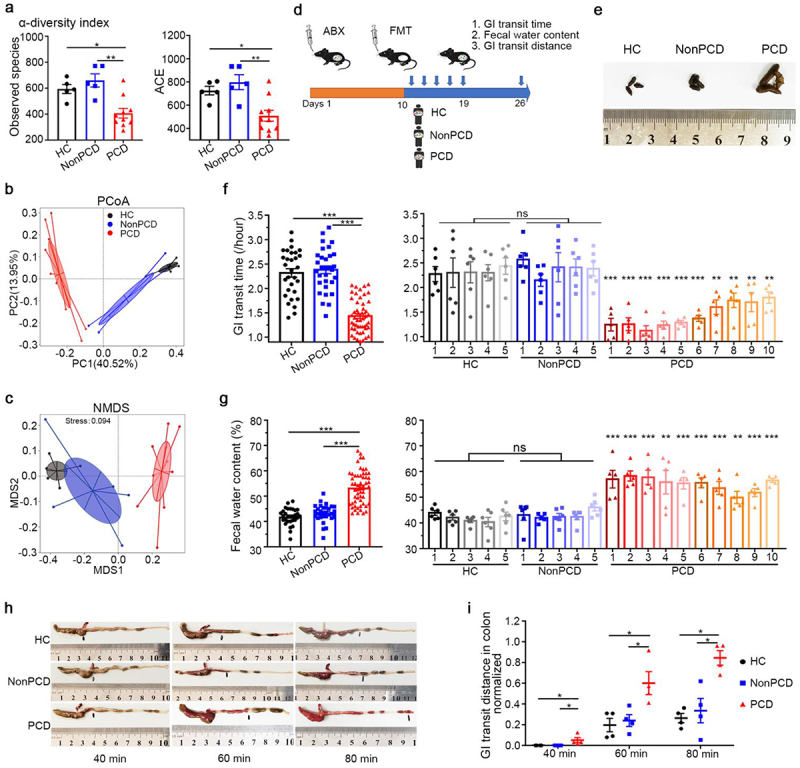
Pseudo-germ-free mice exhibited diarrhea phenotypes after transplanted with PCD gut microbiota. (a) Attenuated bacterial richness and evenness by reduced α-diversity (observed species and ACE) in PCD patients, compared to HC and NonPCD patients. (b) Nonmetric multidimensional scaling (NMDS) plot with cluster indicating differential microbial distributions among three groups. (c) Principal coordinate analysis (PCoA) plot with cluster demonstrating difference in microbial structure among these groups. (d) Experimental design showing the process of humanized microbiome to pseudo-germ-free mice (n = 6 per group): after gavaged with antibiotic cocktail (ABX) for consecutive 10 days, pseudo-germ-free mice were transplanted with fecal microbiota from 20 individuals including 5 HC, 5 NonPCD patients and 10 PCD patients for 5 times, and 7 days after colonization, diarrheal indexes were measured. (e) Representative photograph displaying remarkably changed fecal appearance of PCD mice (n = 3/group). (f) Whole gastrointestinal transit time in three groups (left panel) and each donor (right panel, n = 5–6), respectively. (g) Fecal water content in three groups (left panel) and individuals (right panel, n = 5–6), respectively. (h) Representative photographs for peristalsis distance of carmine solution in colon of each group 40 min, 60 min and 80 min after the gavage, respectively. (i) Quantification of the peristaltic distance of each group at three timepoints, normalized by the length of colon (n = 12/group). Data are shown as mean ± SEM; *p < .05, ** p < .01, *** p < .005.

Initially, 20 fecal samples from patients were selected and subjected to 16S rRNA sequence. Decreased α-diversity index (Observed species and ACE index) indicated reduced bacterial richness and evenness in PCD patients, compared to HC and NonPCD patients (Figure 1a; Figure S1a). Then, obvious discriminations in microbial composition among three groups were revealed by the nonmetric multidimensional scaling (NMDS) model and Principal coordinate analysis (PCoA) (Figure 1b and c), and this was also confirmed by unweighted pair-group method with arithmetic means (UPGMA) tree (Figure S1b). The dramatically differential fecal microbiota in PCD patients was consistent with our previous results.^8^

To investigate whether bacterial changes in PCD patients contribute to diarrhea, humanized gut microbiome mice model (n = 6/donor) was constructed by fecal microbiota transplantation from 20 donors, including 5 HC, 5 NonPCD patients and 10 PCD patients. The experimental process was displayed in Figure 1d. Dramatic difference of fecal pellets appearance was observed in PCD mice, compared with these from HC and NonPCD (Figure 1e). The longer and softer feces intuitively implied changed gastrointestinal functions in PCD mice.^16^ Thereafter, carmine red in methylcellulose solution and fecal water content were applied to evaluate gastrointestinal motility and secretion. The shorter gastrointestinal transit time in PCD mice (Figure 1f, left panel) indicated augmented intestinal motility, without the length change of whole gastrointestinal tracts (Figure S1c), in contrasted to HC and NonPCD mice. These phenotypes were more obvious in PCD 1, 2, 3, 4, 5 mice (Figure 1f, right panel). Then, higher absolute fecal water content was found in PCD mice (Figure 1g). These *in vivo* results demonstrated that altered gut microbiota from PCD patients contributed to diarrhea.

Then, we sought to investigate which segment of gut contribute to enhanced gastrointestinal motility. First, shortened gastrointestinal transit time and elevated fecal water content (Figure S2a and b) were induced again by mixture PCD fecal bacteria. Then, mice were sacrificed at 4 timepoints (30, 40, 60, 80 min, respectively) after carmine solution gavage. Carmine was still transited in small intestine 30 min after gavage and normalized gastrointestinal transit distance was compatible among three groups (Figure S2c and d). At the timepoint of 40 min, the marker was squeezed through ileocecal valve and transited to ascending colon in PCD mice, but it just reached ileocecal valve in HC and NonPCD mice (Figure 1h). Similarly, red pellets were propelled faster in colon of PCD mice than HC and NonPCD mice at timepoint of 60 min and 80 min after gavage, respectively. The quantified results showed that the colonic propulsion of PCD mice was dramatically greater than HC and NonPCD mice (Figure 1i). These findings suggested that PCD fecal bacteria primarily facilitated colonic motility, rather than influenced the motility of small intestine.

### 2.2. Tryptophan metabolism was significantly enriched in bacterial predicted function of PCD mice.

**Figure 2.**
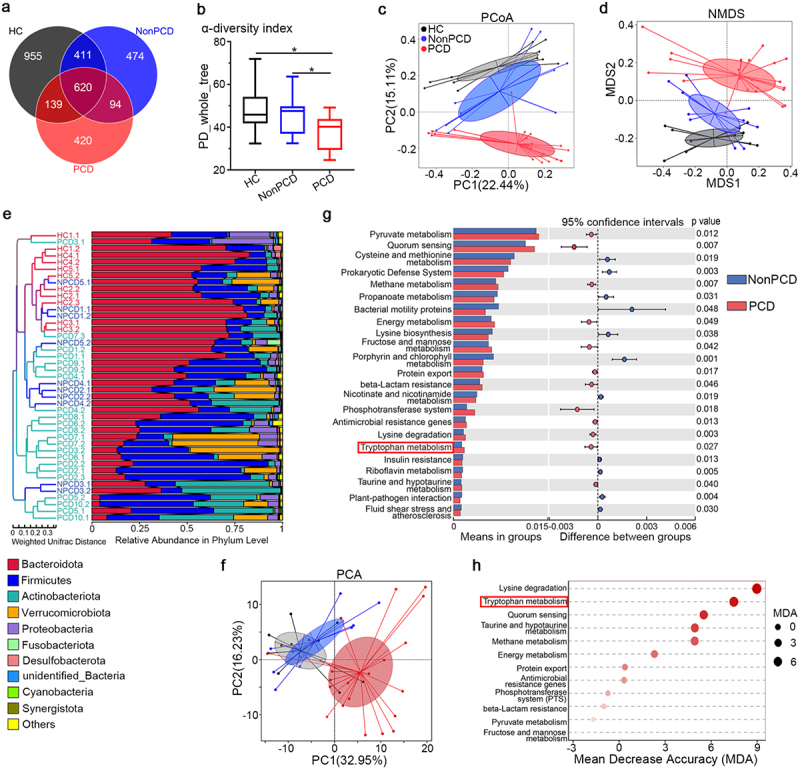
Tryptophan metabolism in bacterial predicted functions was enriched in PCD. (a) Overlaying Venn diagram indicating fewest operational taxonomic units (OTU) of gut microbiota in PCD mice, compared to HC and NonPCD mice. (b) Attenuated bacterial richness and evenness by reduced α-diversity (PD-whole-tree) in PCD mice, n = 11 for HC, n = 10 for NonPCD and n = 22 for PCD mice. Data are presented in Box plot as Mix to Max. * p < .05. (c) Microbial distributions by NMDS plot with cluster in mice among three groups. (d) Differences of microbial structure by PCoA plot with cluster in three grouped mice. (e) Unweighted pair-group method with arithmetic means (UPGMA) tree based on weighted unifrac distance showing microbial differences among each individual mice (left panel) and relative abundance of top 10 bacteria in phylum level (right panel). (f) Principal coordinate analysis (PCA) showing difference of bacterial predicted functions in level 3 by PICRUSt among three groups. (g) Histograph of bacterial predicted functions in level 3 of KEGG pathway analyzed by Tax4Fun showing differentially abundant functions with significance between NonPCD and PCD mice. (h) Ranking of microbial predicted functions in KEGG pathway enriched in PCD mice according to their mean decrease accuracy (MDA) in unsupervised RandomForest analysis.

In light of the irritated colonic propulsion of PCD mice, bacteria in colonic luminal contents were analyzed. The overlapping operational taxonomic units (OTU) Venn diagram displayed that PCD mice possessed the fewest OTU (Figure 2a), compared to NonPCD and HC mice. Declined microbial diversity and higher beta distance by weighted Unifrac (Figure 2b; Figure S3a and b) were present in PCD mice. The NMDS and PCoA plot revealed clear segregations of bacterial structure among groups (Figure 2c and d), which also consisted with UPGMA result (Figure 2e). Bacterial top 10 phylum abundance in individuals and groups showed over-presented *Firmicutes, Verrucomicrobiota* and *Proteobacteria*, but under-presented *Bacteroidota* in PCD mice (Figure 2e; Figure S3c and d). These results indicated huge differences of bacterial composition in PCD mice.

Additionally, PICRUSt and Tax4Fun^17^ were employed to predict bacterial metagenome functions in metabolism and ecology. Principal coordinate analysis (PCA) analysis indicated divergent cluster of bacterial predicted functions in KEGG pathway level 3 among three groups (Figure 2f), and tryptophan metabolism et al were significantly enriched in PCD mice (Figure 2g). After that, these enriched microbial functions were ranked by unsupervised RandomForest analysis, and lysine degradation, tryptophan metabolism and quorum sensing were highlighted (Figure 2h). Whereas, significance in tryptophan metabolism (Figure S3e) among groups, but not lysine degradation (Figure S3f) was achieved. Since that quorum sensing functions as vital reflector for the inter-relationships among bacteria in microbial system^18^ and its potential role was also emphasized in PCD (Figure 2g; Figure S3g). The remarkably decreased co-occurrence network (P_fdr_<0.05, r > 0.7) among genera in PCD mice (Figure S4) as well as our previous results in patients^8^ indicated that weakened bacterial ecology might be the consequence of decreased microbial diversity. Therefore, we hypothesized that some molecules from tryptophan may underlie accelerated colonic peristalsis in PCD mice.

### 2.3. Elevated serotonin biosynthesis and overexpression of 5-HT receptor in colon of PCD mice.

**Figure 3.**
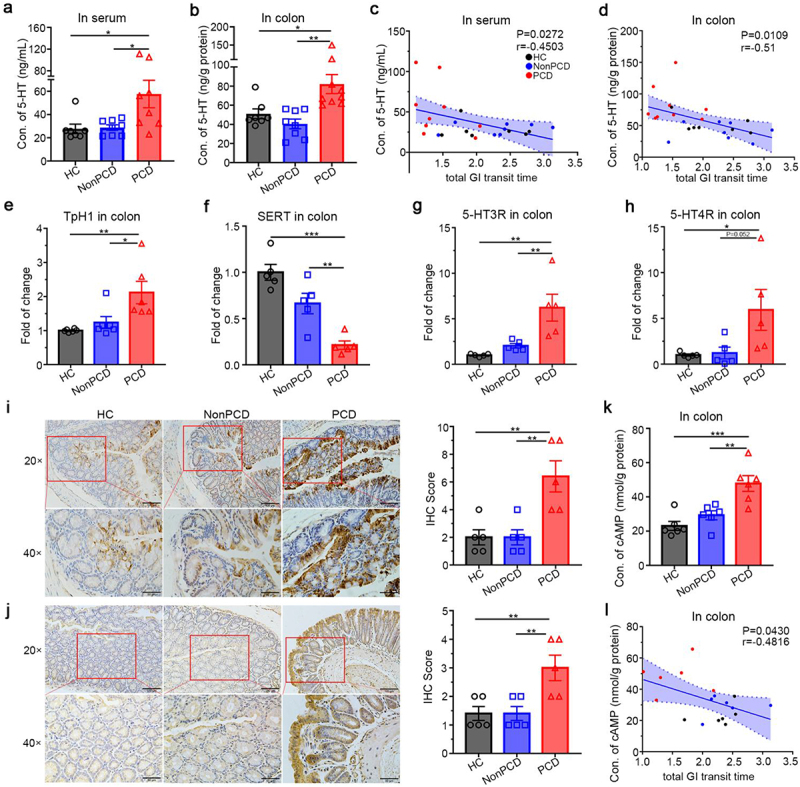
PCD fecal microbiota causes elevated serotonin level and overexpression of 5-HT receptors (5-HTR) in colon of PCD mice. (a) Concentration of 5-HT in serum of three grouped mice, n = 7–8. (b) Quantification of colonic 5-HT level normalized by total protein in mice (n = 7–9/group). (c) Negative correlation with significance between serum 5-HT and gastrointestinal transit time among these groups, r = −0.4503. (d) Significantly negative correlation between colonic 5-HT and gastrointestinal transit time among three groups, r = −0.51. (e) Relative expression of *TPH1* to *β-actin* in colon of mice (n = 6) (f) Colonic expression of *SERT* to *β-actin* among three groups (n = 5). (g, h) Colonic expressions of 5-HT receptors relative to *β-actin* in mice, 5-HT3R (g) and 5-HT4R (h), n = 5 for each group. (i, j) Representative IHC photographs (left panel) of strained 5-HT3R (i) and 5-HT4R (j) in colon and the IHC score (right panel), n = 5/group, upper panel scale bar 100 μm and lower panel 50 μm. (k) Accumulated cAMP level in colon of PCD mice, compared to HC and NonPCD mice, n = 6/group. (l) Negative correlation with significance between colonic cAMP and total GI transit time. Data are expressed in mean ± SEM; * p < .05, **p < .01.

Since 5-hydroxytryptamine (5-HT), also named serotonin, is a fundamental metabolite of tryptophan, which conventionally stimulates intestinal propulsion and segmentation motility.^19^ The critical role of indigenous fecal microbiota on controlling 5-HT biosynthesis is well documented.^15^ Thus, we first quantified its serum and colon level among three grouped mice and PCD mice displayed approximately doubled circulating and in situ 5-HT (Figure 3a and b), compared to HC and NonPCD, besides, they were also negatively correlated with gastrointestinal transit time (Figure 3c and d). As regards this finding, we next compared intestinal enterochromaffin cells (ECs) among three groups, where peripheric serotonin is almost generated. Whereas, compatible colonic mRNA expression of chromogranin A and immunohistochemical (IHC) score in protein level (Figure S5a and b) among groups implied that elevated 5-HT wasn’t derived from over-proliferation of ECs.

Peripheric serotonin metabolism comprising biosynthesis by tryptophan hydroxylase 1 (Tph1) in ECs, reuptake to enterocytes by serotonin selective reuptake transporter (SERT), and eventually catabolism by monoamine oxidase-a (Maoa).^20^ Then, these critical intermediates were further evaluated, and upregulated Tph1 with downregulated SERT (Figure 3e and f) in colon of PCD mice were found, compared to HC and NonPCD, but no significance of colonic Maoa was achieved (Figure S5c). Accordingly, 5-HT metabolism was also examined in small intestine, neither 5-HT level, nor its vital metabolic genes were incompatible among groups (Figure S6a-c). Circulating 5-HT might originate from its overproduction in colon of PCD mice (Figure S6d). Therefore, above results supported that overproduction and down-reuptake of 5-HT accounted for elevated colonic 5-HT in PCD mice, which were regulated by PCD fecal bacteria.

The regulatory role of serotonin on intestinal motility is mediated by 5-HT receptors (5-HTR), of whose family, 5-HT3 and 5-HT4 subtypes in intestinal epithelium and submucosal neurons were primarily responsible for gastrointestinal propulsion and secretion.^19^ Initially, both colonic mRNA expressions were quantified and our results demonstrated that 5-HTRs were simultaneously overexpressed in colon of PCD mice, in contrast to that of HC and NonPCD mice (Figure 3g and h), which were also confirmed by increased IHC score (Figure 3i and j). Intriguingly, those elevated 5-HTRs were mainly located to superficial mucosal epithelium of colons. The 5-HT4R is a G-protein-coupled-receptor and increased downstream signaling cAMP reflected its activation,^21^ doubled cAMP level in colon and negative association between cAMP level with gastrointestinal transit time (Figure 3k and l) indicated its activated state. However, neither expressions of 5-HTRs, nor cAMP level in small intestine were significantly changed among three groups (Figure S6e-g), which showed invariant quantity and nonactivated states of 5-HTRs in small intestine. Thus, elevated colonic 5-HT might be a key actuator in accelerating colon motility in PCD mice, which might be mediated by overexpressed epithelial 5-HT3R and 5-HT4R.

### 2.4. The pro-motility effects of PCD fecal microbiota were serotonin-mediated and 5-HT receptors dependent.

**Figure 4.**
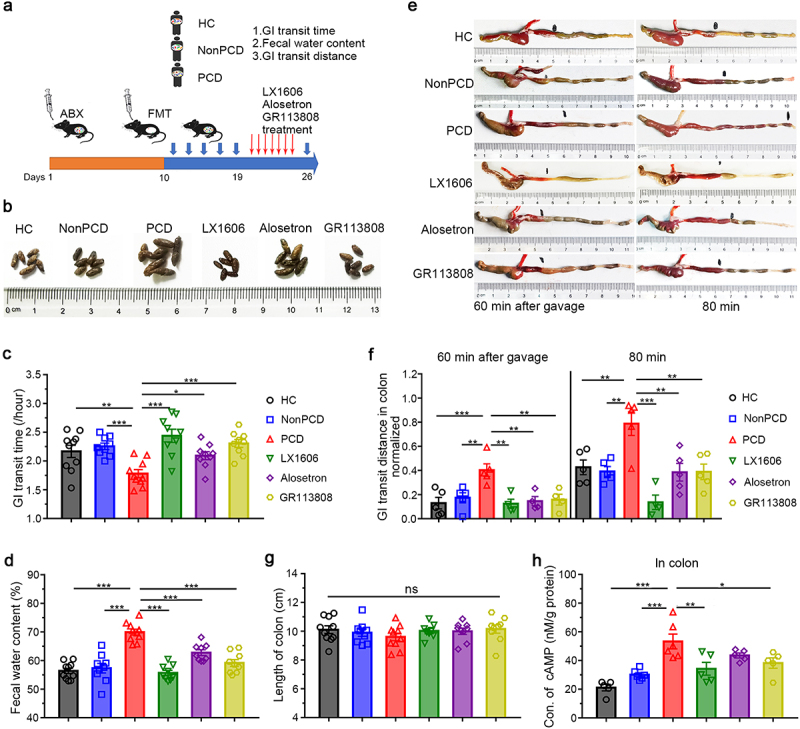
Inhibited synthesis of 5-HT and selective blockade of 5-HT receptors significantly ameliorated diarrheal phenotypes in PCD mice. (a) Experimental design showing the process of humanized microbiome to pseudo-germ-free mice and drugs administration: after construction of humanized microbiome mice, LX1606 (100 mg/kg), alosetron and GR113808 (1 mg/kg) were intraperitoneally injected to mice transplanted with fecal microbiota of PCD patients daily for consecutive 7 days. (b) Photograph displaying fecal appearance among six groups (n = 6/group). (c) Whole gastrointestinal transit time and (d) Fecal water content in these groups (n = 10). (e) Representative photographs for colonic motility of each group 60 and 80 min after carmine solution gavage, respectively. (f) Quantification of normalized peristalsis distance in colon of each group (n = 5) at two timepoints (60 min and 80 min). (g) Colonic length of each group (n = 10). (h) Concentration of cAMP level in colon among six grouped mice, n = 5–6. Data are displayed as mean ± SEM; * p < .05, **p < .01, *** p < .005, ns for not significant.

To boost the confidence of serotonin-stimulated and 5-HTRs-mediated hypermotility in PCD mice, we employed classical Tph1 inhibitor Telotristat ethyl (LX1606)^22^ and 5-HTR antagonists alosetron,^23^ GR113808^24^ for 5-HT3R and 5-HT4R blockade, respectively. After fecal microbiota transplantation, LX1606, alosetron and GR113808 were administrated to PCD mice (Figure 4a). The feces of treated mice resembled to those of HC and NonPCD mice, but significantly differ from PCD mice in appearance (Figure 4b). Subsequently, gut motility was assessed and we found LX1606, alosetron and GR113808 all could extend this index significantly (Figure 4c), compared to untreated PCD mice. Besides, the elevated fecal water content in PCD mice was partially reserved upon drugs’ administration (Figure 4d).

To assess the colonic motility among six groups, we retested the propulsive ability of colon 60 min and 80 min after carmine solution gavage. PCD mice exhibited longest transit distance in colon at both timepoints, compared to HC and NonPCD; but it reduced to normal level upon drugs’ administration (Figure 4e and f), while there was no significance in length of colons among groups (Figure 4g). These results indicated significant alleviation of accelerated colonic motility in treated mice. Finally, declined cAMP in treated mice indicated sufficient blockade of 5-HTR (Figure 4h). Taken together, our results confirmed the responsibility of cumulated 5-HT for colonic hyperperistalsis, and demonstrated the therapeutic effects of specifical 5-HTRs blockade on PCD mice.

### 2.5. Microbial metabolites from PCD mice and patients induced 5-HT over-production

**Figure 5.**
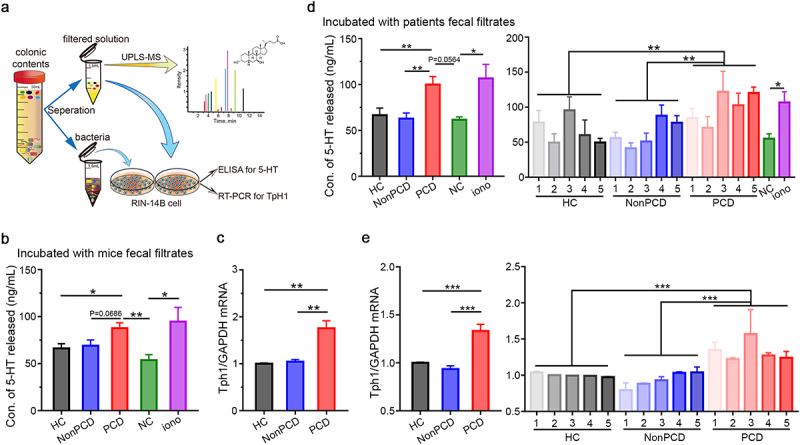
Microbial metabolites from PCD mice and patients stimulated 5-HT biosynthesis in RIN14B cell line. (a) Schematic diagram showing the procedure of microbial metabolites-induced 5-HT overproduction. Fecal pellets were dissolved, filtrated, and centrifuged, then, 120 μL/mg washed bacteria and 0.1 g/mL filtrated solution were incubated with RIN-14B cell, respectively, and UPLC/MS was applied to determine BAs metabolism in feces. (b) Excessive serotonin level released in RIN-14B cell supernatants stimulated by fecal filtrates from PCD mice, ionomycin was applied for positive control, n = 3–6. (c) Elevated mRNA expression of intracellular TpH1 in RIN-14B cells after exposed to fecal filtrates from PCD mice, n = 6. (d) Serotonin level released in RIN-14B cell supernatants among three grouped donors (left panel) and 15 individuals (right panel). (e) Elevated mRNA expression of intracellular TpH1 in RIN-14B cells after exposed to fecal filtrates from PCD patients (left panel) and relative expression of TpH1 in RIN-14B cells, when exposed to fecal filtrates from 15 individuals (right panel). Data are displayed as mean ± SEM; NC normal control; * p < .05, **p < .01, *** p < .005.

To identify the specific contribution of gut microbiota for serotonergic effects, RIN14B cells were incubated with washed fecal bacteria and filtrated solution (Figure 5a) separately. The compatible 5-HT concentrations in RIN14B cell supernatants co-cultured with washed bacteria and intracellular Tph1 expressions (Figure S7a and b) among three groups indicated the impotency of bacteria in stimulating 5-HT release directly. Nevertheless, when exposed to filtered fecal solutions from PCD mice, excessive 5-HT level with significance was synthesized (Figure 5b), accompanied with significantly elevated Tph1 expression (Figure 5c), in contrast to HC and NonPCD. Similarly, these experiments were replicated with washed bacteria and fecal filtrated solutions from 15 donors. Neither 5-HT overproduction in RIN14B, nor the intracellular Tph1 expression could be induced by gut microbiota from 5 PCD individuals (Figure S7c-f). However, when exposed to subjects’ fecal filtrated solutions, the 5-HT concentrations in RIN14B cell supernatant (Figure 5d), as well as the expression of Tph1 in RIN14B cells (Figure 5e) increased significantly in grouped PCD patients and individuals. In conclusion, these results proved the responsibility of microbial metabolites for serotonergic effects.

### 2.6. Gut microbial secondary bile acid metabolites stimulated serotonin generation

**Figure 6.**
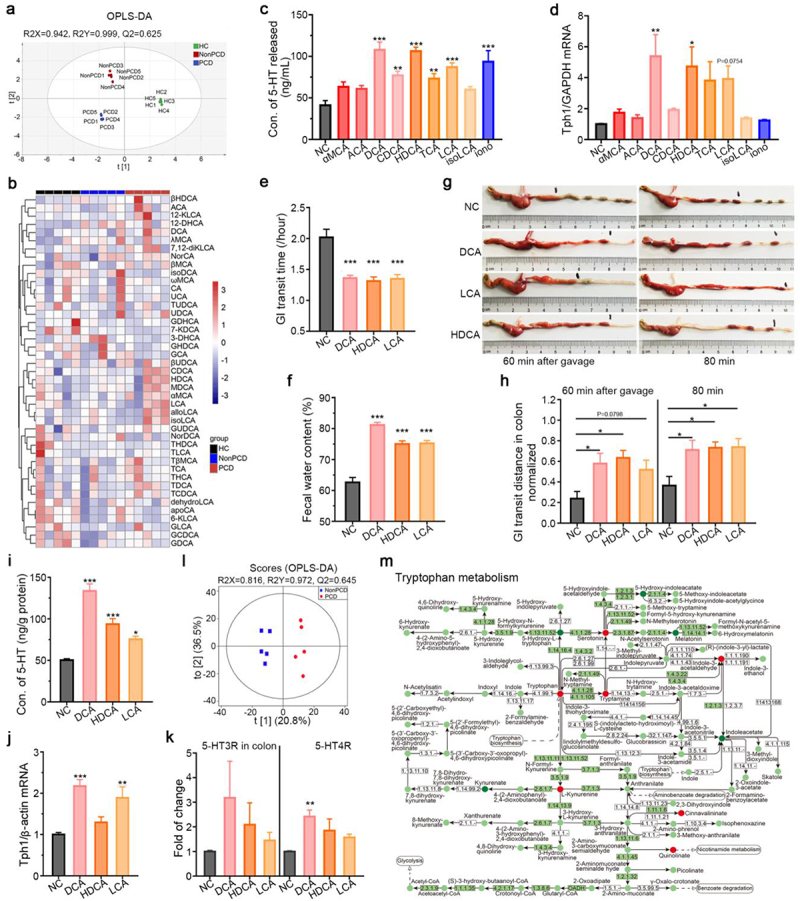
Overproduction of 5-HT induced by microbial bile acid metabolites. (a) Distinction in fecal bile acid metabolism by the Orthogonal Projections to Latent Structures Discriminant Analysis (OPLS-DA) model among three grouped mice, n = 5. (b) Heatmap showing difference of 43 individual BAs in the feces of three grouped humanized gut microbiota mice. (c) Level of 5-HT in RIN-14B cell supernatant when exposed to bile acids metabolites: α-muricholic acid (50 μM), Allocholic acid (50 μM), deoxycholic acid (25 μM), chenodeoxycholic acid (50 μM), hyodeoxycholic acid (250 μM), taurocholic acid (250 μM), lithocholic acid (50 μM), isolithocholic acid (50 μM), n = 3–8. (d) Relative expression of *TPH1*/ *GAPDH* in RIN-14B cells when exposed to these BAs metabolites, n = 3–6. (e) Secondary bile acids (DCA, HDCA and LCA) expedite GI transit in vivo, n = 10–12. (f) Fecal water content in mice treated with these secondary BAs (DCA, HDCA and LCA), n = 10–12. (g) Representative photographs showing the in vivo pro-motility effects of these BAs. (h) Extended normalized peristalsis distance in colon by these BAs 60 min and 80 min after carmine solution gavage, n = 5–6. (i) Concentration of colonic 5-HT in mice treated with these BAs, n = 9–11. (j) Relative expression of *TPH1* to *β-actin* in colon of mice treated with these BAs, n = 5. (k) Elevated expressions of 5-HT receptors to *β-actin* in colon of BAs-treated mice, n = 5. (l) OPLS-DA plot revealing discrimination of fecal tryptophan metabolism between NonPCD and PCD patients, n = 5. (m) KEGG pathway map showing differential metabolites of tryptophan metabolism in feces of NonPCD and PCD patients, red circles denoted upregulated metabolites and green circles downregulated. Data are displayed as mean ± SEM; NC normal control; * p < .05, **p < .01, ***p < .005.

In accordance, we have elucidated altered fecal BAs metabolism in PCD patients previously,^8^ but whether changed BAs metabolites contribute to PCD through 5-HT remains unknown. Therefore, fecal BAs metabolism among recipient mice was determined by UPLC/MS (Figure 5a). The Orthogonal Projections to Latent Structures Discriminant Analysis (OPLS-DA) model indicated dramatically distinctive fecal BAs profiling in PCD mice (Figure 6a). When analyzing individual BAs metabolites, 11 candidates such as deoxycholic acid (DCA), hyodeoxycholic acid (HDCA) and lithocholic acid (LCA) enriched, but 6 such as cholic acid (CA) reduced in PCD mice (Figure 6b), the quantified results were shown in Figure S8b and supplementary table S4. Then, the serotonergic effects of these specific metabolites enriched in PCD mice were tested in RIN14B cells. Of these 8 BAs, DCA, HDCA and LCA could double 5-HT level significantly, but taurocholic acid (TCA), chenodeoxycholic acid (CDCA), isoLCA and α-muricholic acid (αMCA) only increase it slightly (Figure 6c). Furthermore, cumulated 5-HT correspond to overexpressed Tph1 (Figure 6d) implied the obvious serotonergic effects of DCA, LCA and HDCA. Then, the diarrhea-inducible role of these secondary BAs (DCA, LCA and HDCA) were tested *in vivo* and we found that they could shorten gastrointestinal transit time for 0.6-fold and elevated fecal water content for 1.3-fold (Figure 6e and f). Besides, the pro-motility effects on colon of these BAs were found by intracolonic injection of BAs (Figure 6g and h). Then, *in vivo* serotonergic effects of them were found by increased colonic 5-HT level and overexpressed *TPH1* in BAs-treating mice (Figure 6i and j), intriguingly, over-expressions of 5-HT receptors were also found in colon of BAs-treated mice (Figure 6k). Therefore, microbial secondary BAs could stimulate 5-HT production *in vitro* and *in vivo*.

To further confirm BAs-induced overproduction of serotonin in PCD patients, we profiled fecal BAs metabolism and tryptophan metabolism, and found overabundant BAs metabolites in feces of PCD patients, compared to HC and NonPCD individuals (Figure S8e-g). The OPLS-DA with good ability displayed an obvious segregation in tryptophan metabolism between NonPCD and PCD patients (Figure 6l). Then, KEGG enrichment map on tryptophan metabolism pathway to depict the integrative interactions among these metabolites was applied, and serotonin metabolic pathway including tryptophan, serotonin and tryptamine was evidently enriched in feces of PCD patients (Figure 6m). Besides, the total fecal 5-HT amount was accumulated with marginal significance in PCD patients (Figure S8h), which suggested active serotonin biosynthesis in colon.

Interestingly, these BAs metabolites tested with serotonergic effects were secondary BAs, whose metabolism depended heavily on fecal microbes.^13^ Thus, we analyzed microbial differences among humanized gut microbiome mice, *clostridium spp*. and *unidentified-Lachnospiraceae* encoding 7-dehydroxylases, vital microbial enzymes for BAs synthesis,^25^ were found overabundant in PCD mice (Figure S9a-d), as well as in feces of PCD patients (Figure 9e and f). Thus, cumulative fecal secondary BAs possibly resulted from overabundant BAs-transforming bacteria in PCD gut microbiota.

### 2.7. Blocking BAs conjugated TGR5/TRPA1 receptors significantly alleviated PCD gut microbiota-induced diarrhea

**Figure 7.**
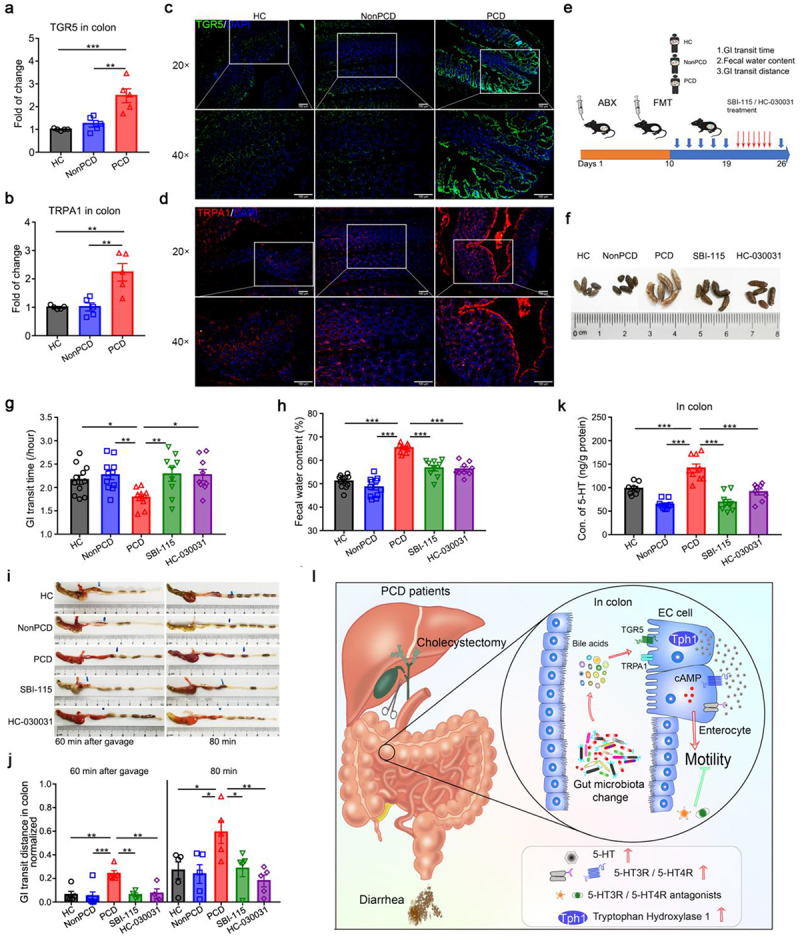
PCD fecal microbiota induce diarrhea via TGR5/TRPA1 signaling pathway. (a, b) Elevated expressions of *TGR5* (a) and *TRPA1* (b) to *β-actin* in colon of mice colonized with PCD gut microbiota, n = 5. (c, d) Representative IF photographs of strained TGR5 in green (c) and TRPA1 in red (d) in colon of humanized gut microbiome mice, upper panel scale bar 100 μm and lower panel 50 μm, nucleus were stained with DAPI in blue. (e) Experimental design showing the process of humanized microbiome to pseudo-germ-free mice (n = 10 per group) and SBI-115 (15 mg/kg) and HC-030031 (150 mg/kg) administration: after construction of humanized microbiome mice, these drugs were gavaged to PCD mice daily for consecutive 7 days. (f) Representative photograph displaying fecal appearance in 5 grouped mice (n = 5). (g) Pro-motility effects of PCD microbiota were reversed by TGR5 antagonist (SBI-115) and TRPA1 selective inhibitor (HC-030031), n = 9–11. (h) Elevated fecal water content of PCD mice were lowered by SBI-115 and HC-030031, n = 9–11. (i) Representative photographs of colon displaying the alleviating effects of SBI-115 and HC-030031 on colonic motility. (j) Quantification of normalized colonic motility in 5 grouped mice, n = 4–6. (k) Colonic levels of 5-HT among 5 groups, n = 8–9/group. Data are displayed as mean ± SEM; * p < .05, **p < .01, ***p < .005. (l) Schematic diagram of diarrhea-inducible effects of PCD fecal microbiota. PCD fecal microbiota could induce diarrheal phenotypes mainly through accelerating colonic motility, and overabundant colonic 5-HT level is identified as the critical factor for increased peristalsis due to its excessive biosynthesis and declined reuptake by PCD microbiota. Furthermore, altered BAs metabolites by PCD gut microbiota leads to overproduction of colonic 5-HT via TGR5/TRPA1 signaling pathway. Finally, 5-HT receptors (5-HT3R and 5-HT4R) were found overexpressed and selective blockade of them could significantly ameliorate microbiota-induced diarrhea by corresponding antagonists.

DCA, LCA and HDCA are discovered as specific agonists for G protein-coupled bile acid receptor 1 (GPBAR1), or TGR5,^26,27^ and transient receptor potential ankyrin 1 (TRPA1) enables ECs secrete 5-HT to regulate gastrointestinal motility after TGR5 activation.^28,29^ Therefore, to verify the diarrhea-inducible roles of bacterial BAs metabolites in PCD, we selectively blocked these two receptors by SBI-115 and HC-030031.^30,31^ First, we quantified the relative expressions of mRNA for them and found both of them doubled in the colon of PCD mice, compared to that of HC and NonPCD mice (Figure 7a and b). Then, *in situ* immunofluorescence (IF) on TGR5 (green) and TRPA1 (red) was applied and elevated fluorescence intensity of them was observed in colon of PCD mice (Figure 7c and d). These results indicated that over-expressed TGR5 and TRPA1 possibly mediated BAs-induced diarrhea by PCD gut microbiota.

After that, we employed TGR5 antagonist SBI-115 and TRPA1 inhibitor HC-030031 in PCD mice (Figure 7e). The feces of treated mice resembled to those of HC and NonPCD mice, but significantly differ from PCD mice (Figure 7f). Then, decreased gastrointestinal transit time was reversed upon these drugs administration in PCD mice, along with lower fecal water content in treated-mice, in contrast to untreated PCD mice (Figure 7g and h). Besides, the augmented colonic motility of PCD mice was significantly alleviated by SBI-115 and HC-030031 (Figure 7i and j). Finally, when determining the blocking effects on colonic 5-HT level, we found elevated 5-HT concentration in colon of PCD mice declined in half upon drugs administration (Figure 7k). Therefore, these evidences proved the pro-motility roles of bacterial secondary BAs metabolites in PCD and implied therapeutical efficacy of TGR5 antagonist and TRPA1 inhibitor on PCD.

## 3. Discussion

Our study first reveals the diarrhea-inducible role of altered fecal microbiota in post-cholecystectomy diarrhea (PCD) (Figure 7l). Although we have previously revealed the correlation between changed gut microbiota and PCD,^8^ nevertheless, whether altered gut microbiota is merely a consequence of PCD *per se* or a contributing factor remains unsupported. Herein, we solved this through fecal microbiota transplantation to pseudo-germ-free mice. Enhanced gastrointestinal motility and increased fecal water content in PCD mice accorded with diarrheal phenotypes,^8^ besides, colonic hypermotility by PCD fecal microbiota also consisted with clinical observations that increased colonic propulsion in PCD patients.^4^ The deleting efficiency on gut microbiota by ABX in pseudo-germ-free mice model was exhibited in our previous article;^32^ and after fecal microbiota transplantation, the similarity of bacterial composition between patients and recipient mice indicated the success and good ability in constructing PCD mice model.^25^ Thus, bacterial alteration in PCD functions as an important pathogenesis for postoperative diarrhea. Similar as IBD and IBS diarrhea (IBS-D), PCD patients exhibited increased defecation frequency and fecal water content, but the pathophysiological mechanisms of PCD differed from IBD and IBS-D greatly. Numerous articles have identified gut bacterial dysbiosis as crucial pathogenic factor for IBD and IBS-D,^33^ this study added evidence of changed gut microbiota for PCD. However, differences on bacterial structure among these diseases require more investigations.

Then, in analyzing the mechanism of PCD fecal bacteria-induced diarrhea, colonic serotonin receives much attention for the vital clue that enriched tryptophan metabolism in predicted bacterial functions by KEGG analysis. As an important metabolite of tryptophan, 5-HT is known for its vital role in maintaining gastrointestinal motility;^19^ and it’s well appreciated that 5-HT tightly correlates with clinical gastrointestinal disorders, for example, elevated plasma 5-HT in IBS diarrhea patients.^33^ Initially, elevated 5-HT level in PCD mice and alleviated hyperperistalsis in LX1606-treated PCD mice confirmed our hypothesis that 5-HT might be a critical executant in inducing PCD. Furthermore, to figure out the inner mechanism of serotonin metabolism, increased generation by Tph1 and repressed reuptake by SERT were found in colon of PCD mice. Upregulating Tph1^15^ and downregulating SERT^34^ by fecal bacteria have been mentioned before, therefore, both of them were supposed to be responsible for cumulated 5-HT level.^35^

Importantly, the highest level of 5-HT in colon and indiscrimination in small intestine among three groups indicated that its production is stimulated by PCD fecal bacteria, with the fact that colon resident gigantic amounts of bacteria than small intestine.^7^ Regulatory repertoires of bacteria in host physiology depend on their metabolic functions and the stimulating effects of gut microbiota on serotonin production is mediated by microbial metabolites.^15,36^ Bile acid diarrhea is universally recognized as a pathogenic factor of PCD and we disclosed the relations of altered fecal BAs with PCD.^8^ Thus, fecal BAs metabolites were profiled, next the serotonergic effects of specific bacterial BAs metabolites were investigated in RIN14B cell line, a model of ECs cell function. Fortunately, three efficient candidates, DCA, HDCA and LCA were identified. The stimulating effect of DCA on 5-HT production has been stated,^15^ moreover, we first testified the *in vitro* and *in vivo* serotonergic effects of another two secondary BAs, LCA and HDCA. LCA doubled Tph1 expression, but only promoted 5-HT level for 1.36-fold, less than 1.86-fold of HDCA, this interesting result attributed to overexpressed SERT by LCA. Apart from the already revealed pro-motility effects of DCA and LCA,^30^ we initially obtained similar result of HDCA. Therefore, these results provided rational explanation that cumulative secondary BAs metabolites induce colonic hyperperistalsis via stimulating serotonin generation.

Intriguingly, in analyzing fecal BAs metabolism, slightly overabundant secondary BAs was found in feces of PCD mice, with the compatible primary BAs concentrations (Table S5). Besides, higher ratio of secondary BAs to primary (Table S5) indicated bacterial activity of biotransformation. Secondary BAs, especially DCA, LCA and HDCA, relies heavily on microbial diversifications, such as dehydroxylation.^13,36^ Then, *Clostridium spp*. and *unidentified Lachnospiraceae*, which bear 7-dehydroxylation for DCA, LCA and HDCA biotransformation^37,38^ were overabundant in PCD bacterial composition. Furthermore, other genera of *Lachnospiraceae* were also highlighted. However, this study is limited in elaborating the causal relationships of specific gut bacteria and their encoding BAs, such causality requires more experiments to further confirm. Indeed, it is fully demonstrated that secondary BAs were transformed from primary BAs with the help of fecal bacteria,^13,37^ and two microbes known to bear 7-dehydroxylation were found overabundant, which provided reasonable interpretation for excessive secondary BAs.

In verifying the serotonin-mediating diarrhea by PCD fecal bacteria, we employed LX1606, alosetron and GR113808 to blocking 5-HT from different aspects. Since that the regulatory repertoires of 5-HT in gastrointestinal peristalsis depend on 5-HT receptors (5-HTR) and two subtypes (5-HT3R and 5-HT4R) are uniquely distributed in gastrointestinal tracts.^39^ The overexpressed 5-HTRs in PCD mice is found complied with accumulated serotonin and could be stimulated by secondary BAs in our study. These two 5-HTRs are gradually accepted as attractive therapeutic targets for diarrhea,^19,40^ and several antagonists are currently developed as efficient drugs for IBS diarrhea, such as 5-HT3R antagonist alosetron.^23,41^ Besides, the clinical efficacy of other 5-HT3R pharmaceutical agents, such as ondansetron and ramosetron, are also substantiated.^41,42^ Likewise, as a selective antagonist for 5-HT4R, GR113808 could inhibit intestinal motility,^43^ which indicate its potential in treating diarrhea. Furthermore, we employed LX1606 to reduce 5-HT biosynthesis and our results demonstrated its efficacy in PCD. LX1606 is accepted to treat augmented bowel movement in patients with carcinoid syndrome,^44^ thus this study also implied its potential application in PCD.

Finally, we explicit the vital parts of TGR5 and TRPA1 in mediating the pro-motility effects of secondary BAs. TGR5 and TRPA1 distributed on intestinal mucosa serve as critical irritant sensors for luminal secondary BAs and mediate ECs secret excessive 5-HT to promote gastrointestinal propulsion.^29,31,45^ It’s reported that DCA stimulated 5-HT release and promote intestinal propulsion via TGR5 and *tgr5-ko* mice exhibited obvious constipation.^46^ Besides, TRPA1 agonist could promote the contraction of ileum by stimulating 5-HT in ECs.^47^ Initially, both overexpression in colon of PCD mice were revealed in our study. Nevertheless, once they were blocked, colonic hypermotility in PCD mice were significantly alleviated and elevated 5-HT level was reduced to normal. By virtue of the alleviating effects of SBI-115 on LCA-expedited colonic transit,^30^ our results suggested that administration of SBI-115 and HC-030031 efficiently prevent BAs-induced hyperperistalsis via reducing colonic 5-HT level. Therefore, the pro-motility effects of secondary BAs by PCD bacteria are mediated by TGR5/TRPA1 signaling pathway.

In this study, we demonstrated the diarrhea-inducible effects of PCD fecal microbiota and revealed the serotonergic effects of bacterial secondary BAs. Herein, we inhibited this pathway and obtained therapeutical efficacy on PCD by several drugs, which provided new approaches in treating postoperative diarrhea. Therefore, these receptors could be meaningful therapeutical targets for PCD. However, systemic adverse or off-target effects of these antagonists still can’t be neglected despite the rare incidence.^22–24,30,31^ Remodeling patients’ fecal microbiome through fecal microbiota transplantation is currently recognized as an effective procedure for gastroenteric diseases with high safety.^48,49^ The clinical application of fecal microbiota transplantation is being extensively promoted; thus, this may reduce 5-HT biosynthesis from the origin radically and significantly alleviate PCD.

In summary, this study certified the causality of gut microbiota alteration on PCD, and revealed the gut microbiota-fecal BAs metabolites-TGR5/TRPA1-serotonin axis in this process. Therefore, reducing intestinal 5-HT production or blocking selective 5-HTRs might be exploited as new therapeutic modalities for PCD, yet reconditioning gut microbiota of diarrheal patients is more radical.

However, one limitation of this research is that the causal relationships of specific diarrhea-inducing bacteria and corresponding encoding BAs are not identified, and such causality requires advances of *in vitro* culture of gut microbiota. Another is that existing huge gaps between TGR5/TRPA1 activation and cumulative 5-HT level, moreover, the downstream pathways of 5-HTRs-mediated gastrointestinal motility were poorly elucidated. Indeed, more investigations are needed to solve these shortcomings.

## 4. Materials and methods

### 4.1. Participants recruiting and sampling.

Post-cholecystectomy diarrhea (PCD) patients (n = 10) were selected in Minhang hospital, Fudan University according to diarrheal criteria^50,51^ as well as NonPCD patients (n = 5) and healthy controls (HC, n = 5). Clinical characters including age, sex BMI, and months after surgery et al were shown in Table S1. Morning first stool samples were obtained from donors and suspended, then frozen and preserved in −80°C. Details of the inclusion criteria and exclusion criteria for those volunteers were shown in supplemental materials.

### 4.2. Mice and humanized microbiome mice model.

Adult 8-weeks-old C57BL/6 mice were maintained in specific-pathogen-free (SPF) conditions with room temperature of 24°C and humidity. All mice were kept on a 12 h light/dark cycle, and fed standard rodent chow and sterile water *ad libitum*. After 1 week acclimation, mice were gavaged daily with 200 μL antibiotics cocktail (ABX) for consecutive 10 days to construct pseudo-germ-free mice model.^25^ ABX solution contained 50 mg/kg vancomycin, 100 mg/kg neomycin, 100 mg/kg metronidazole, 1 mg/kg amphotericin-B, and 1 mg/mL ampicillin in PBS. Feces from 5 HC, 5 Non-PCD and 10 PCD donors were dissolved with sterile PBS to 0.1 g/mL, then, fecal solutions were filtered in 100 μm filters, centrifuged at 8000 g for 10 min, washed with PBS for three times, and resuspended to 100 mg/mL bacteria solutions. To establish humanized microbiome mice model, 200 μL microbiota suspension from independent individuals (Table S1) were gavaged into ABX-pretreated mice (n = 6/donor) for 5 times every other day. Seven days after colonization, gastrointestinal motility and fecal consistency were measured. Feces before ABX treatment, prior to fecal microbiota transplantation and after FMT were collected and snap-frozen in liquid nitrogen and stored in −80°C refrigerator until use.

### 4.3. Ethics statement.

Clinical and animal ethics in this study was authorized by ethics committee of Minhang Hospital, Fudan University, and written informed consents were obtained from all volunteers.

### 4.4. Carmine red assay for in vivo gastrointestinal motility.

Whole gastrointestinal transit time *in vivo* was detected by applying 6% carmine red solution mingled with 0.5% methylcellulose as reported previously.^52,53^ In brief, mice without fasted were gavaged with 10 μL/g carmine red solution at 9:00 am, and the time till the appearance of first red fecal pellet was recorded. Partial gastrointestinal motility was detected by measuring the length of red marker peristalsis in total gastrointestinal tract at four timepoint-30 min, 40 min, 60 min and 80 min, respectively, after the carmine red solution gavage.^54,55^ Blood samples were collected, and gastrointestinal tract was dissected and measured.

### 4.5. Measurement of fecal water content.

Fecal pellets from humanized microbiome mice 7 days after FMT were collected and weighted, then dried in the oven (105°C) for 24 h and weighted again. Finally, fecal water content was calculated by the equation: (W_wet –_ W_dry_)/ W_wet_×100%.

## 4.6. 16S rRNA sequencing.

Fecal samples from mice and patients were subjected to 16S rRNA to determine gut microbiota.^8^ Total genome DNA were extracted by DNA extraction kit. In the template of microbial DNA, hypervariable regions (V3-V4) of 16S rRNA gene were amplified and processed to sequencing libraries. Sample amplicons were sequenced on an Illumina HiSeq platform (Illumina, MiSeq, USA) and 250 bp paired-end reads were generated. Bioinformatic processing on 16S rRNA data were displayed in Supplementary materials. The 16S rRNA database were all deposited to the public platform of NCBI short-read archive with the BioProject number of PRJNA865451.

### 4.7. Targeted profiling fecal BAs and tryptophan metabolism.

BAs metabolites in feces of mice and patients were profiled as previously reported,^8^ and fecal tryptophan metabolites in patients were profiled. Details of these targeted metabolomics were described in Supplementary methods.

### 4.8. Immunohistochemical (IHC) and IHC score.

The IHC procedure for colonic tissues was described as previous.^56^ Fresh colonic tissues were fixed in 4% paraformaldehyde and the 4 μm paraffin sections were sliced. Then, these sections were deparaffinized, rehydrated, antigen-retrieved, and subjected to 3% H_2_O_2_ solution. After blocking by 3% bovine serum albumin solution, the slides were incubated with diluted Chromogranin A rabbit polyclonal antibody (1:200), HTR3A rabbit antibody (1:200) and HTR4 rabbit polyclonal antibody (1:500), respectively. Then, secondary antibody with horseradish peroxidase was added and diaminobenzidine solution was applied, which was followed by 20% hematoxylin. Finally, the IHC signals of these slices were analyzed and scored by two veteran pathologists according to these scoring criteria.^57^

IHC score was calculated by percentage score multiples intensity score. The standards for intensity score were described as follow: 0 for no staining, 1 for weak staining, 2 for moderate staining and 3 for strong staining. The standards for percentage score were described as follow: positive cells less than 5% was scored 0, 6% to 25% scored 1, 26% to 50% scored 2, more than 50% scored 3.

### 4.9. Immunofluorescence (IF) staining.

Immunofluorescence procedure was performed as previously described,^15^ Fresh colon samples were processed to slides and subjected to immunofluorescence staining. Then, these sections were deparaffinized, rehydrated, antigen-retrieved, and subjected to 3% H_2_O_2_ solution. After blocking by 3% bovine serum albumin solution, the slides were incubated with primary antibody, including anti-GPCR TGR5 antibody (1:500) and TRPA1 polyclonal antibody (1:200). Then, secondary antibody conjugated with fluorescein was added and incubated in dark for 50 min, and nucleus were stained with DAPI. Finally, slides were photographed in different channels, where TGR5 was labeled green and TRPA1 red. After background correction, monochrome images were merged using Photoshop CS6 (Adobe).

### 4.10. RIN-14B cell line and treatment.

The rat pancreas islet tumor cell line RIN14B was cultured in RPMI-1640 with 10% fetal bovine serum and 1% penicillin/streptomycin in humidified condition of 37°C, 5% CO_2_. Feces from recipient mice and donors were suspended at 0.1 g/mL in HBSS supplemented with 2 μM fluoxetine and 0.1% bovine serum albumin, then, the solutions were separated for bacteria and filtrated solution. Feces solutions were centrifuged at 12000 g, 10 min, and the supernatants were filtrated in 0.22 μm filters. Then, the residues were resuspended, filtrated in 100 μm filters, centrifuged at 12000 g for 10 min, washed with HBSS for three times, and diluted into 120 μL/mg with HBSS solution. RIN-14B cells were digested, washed with PBS, seeded into 12-well plate (4 × 10^5^/mL), and cultured for 3 days. Then, cells were incubated with 5 μL bacteria for 30 min and 100 μL fecal filtrated solution for 60 min, respectively. Then, cell supernatants were collected, centrifuged, filtered by 0.22 μm filter and stored in – 80°C until 5-HT measurement, and remaining adherent RIN14B cells were lysed for downstream RNA extraction and RT-PCR.^58^ Normal 5-HT level was considered as normal control (NC) and 15 μM ionomycin was added as positive control.

For metabolites sufficiency experiments, RIN-14B cells were incubated with bile acids metabolites (in 1% DMSO) for 60 min at the indicated concentrations: α-muricholic acid (50 μM), Allocholic acid (50 μM), deoxycholic acid (25 μM), chenodeoxycholic acid (50 μM), hyodeoxycholic acid (50 μM), taurocholic acid (50 μM), lithocholic acid (50 μM), isolithocholic acid (50 μM). 5-HT level in cell supernatants were measured by ELISA and relative expression of intracellular Tph1 to GAPDH were determined by RT-PCR.

### 4.11. BAs metabolites in vivo experiments.

After 1 week acclimation, adult C57BL/6 mice were anesthetized with isoflurane, and intrarectally injected with 200 μL BAs metabolites solutions (DCA, HDCA and LCA 125 mg/kg) by a flexible plastic tube (18-gauge, 3 in) every 12 h for 3 days, besides, control mice were injected with 200 μL PBS. At the last injection, mice were gavaged with carmine solution, 60 min and 80 min later, mice were sacrificed, their colon were photographed and colonic 5-HT levels were measured.

## 4.12. 5-HTR antagonists and Tph1 inhibitor administration.

Alosetron and GR113808 were selected as 5-HT3 receptor (5-HT3R) and 5-HT4R antagonists,^24,59^ and Telotristat ethyl (LX1606) was selected as Tph1 inhibitor.^22^ The humanized microbiome mice model was established again by mixture fecal microbiota transplantation from 5 HC, 5 NonPCD and 5 PCD donors. After the last gavage, PCD mice were intraperitoneally injected daily with 200 μL antagonists (1 mg/kg) in 1% DMSO and LX1606 (100 mg/kg) in 10% DMSO and 5% Tween 20 for consecutive 7 days, and three control groups were injected with 200 μL 1% DMSO. Then, total gastrointestinal transit time and fecal water content were measured in 6 grouped mice, and *in vivo* gastrointestinal motility at 60 min, 80 min timepoints after carmine red solution gavage was determined as described above. All animal experiments were repeated three times independently and n = 9–10 for each group.

### 4.13. TGR5 antagonist and TRPA1 inhibitor administration.

SBI-115 and HC-030031 were employed as selective TGR5 antagonist and TRPA1 inhibitor.^30,31^ The humanized microbiome mice model was established again as described above. After the last gavage of gut microbiota, PCD mice were gavage with 15 mg/kg SBI-115 and 100 mg/kg HC-030031 in PBS daily for 7 times, and control groups were gavaged with 200 μL PBS. Then, total gastrointestinal transit time and fecal water content were measured, and *in vivo* gastrointestinal motility at 60 min, 80 min timepoints after carmine red solution gavage was determined as described above. All animal experiments were repeated three times independently and n = 9–11 for each group.

### 4.14. ELISA for 5-HT and cAMP.

Serotonin levels in different samples were determined by ELISA Kits according to the manufacturer’s protocol, as well as cAMP. Besides, the actual concentration of tissue samples was normalized to total protein content as measured by Bradford assay.

### 4.15. Quantitative real-time PCR.

Total RNA was extracted from mouse gastrointestinal tract or RIN14B cells by RNA-Quick Purification Kit according to manufacturer’s directions. Complementary DNA was reversely transcribed from 1 μg RNA using Prime Script™ RT Master Mix. RT-PCR procedure was performed with QuantStudio^TM^ 7 Flex Real-Time PCR System (Applied Biosystems), and reaction mixture containing 5 μL SYBR™ Green Master Mix, 1 μL cDNA template and 0.1 μL of each specific primer (Table S2). Each sample was repeated for three times and relative expression of each gene was calculated by ΔΔCT method.

### 4.16. Statistics

Data are expressed as the mean ± SEM. GraphPad Prism 8.0 and R 2.15.3 were applied for statistical analyses. Unpaired nonparametric Mann-Whitney t-test was conducted for difference comparisons when comparing two groups, and one-way ANOVA was used to comparing multiple groups. * P < .05, ** P < .01, *** P < .001, and ns for not significant.

## Supplementary Material

Supplemental Material

## Data Availability

The 16S rRNA database were all deposited to the public platform of NCBI short-read archive with the BioProject number of PRJNA865451.https://submit.ncbi.nlm.nih.gov/subs/bioproject/SUB11888187/overview.
