# Supplementary material for: Gut microbiota alteration after cholecystectomy contributes to post-cholecystectomy diarrhea via bile acids stimulating colonic serotonin
Source: Gut Microbes. 2023 Feb 2;15(1):2168101. doi: 10.1080/19490976.2023.2168101 (PMC9897804; doi:10.1080/19490976.2023.2168101)
Supplement: Supplemental Material [file KGMI_A_2168101_SM7517.docx]

**Supplementary materials**

**Gut microbiota alteration after cholecystectomy contributes to post-cholecystectomy diarrhea via bile acids stimulating colonic serotonin**

Supplementary materials include

Supplementary methods

Supplementary figures

Figure S1: Reduced bacterial diversity and changed composition in feces from PCD donors.

Figure S2: Compatible peristaltic motility of small intestine among three humanized gut microbiota mice groups.

Figure S3: Declined gut microbial diversity and altered bacterial structure in recipient mice.

Figure S4: Reduced co-occurrence network among top 100 genera in PCD mice.

Figure S5: Undifferentiated colonic enterochromaffin (ECs) cells and compatible Maoa among three groups.

Figure S6: Invariant 5-HT level with unchanged and inactivated 5-HT receptors in small intestine among three grouped mice.

Figure S7: Bacteria from PCD mice and patients failed to elevated 5-HT level and stimulate Tph1 in RIN14B cells.

Figure S8: Overabundant BAs metabolites in feces of PCD patients.

Figure S9: Overabundant fecal bacteria with metabolic capability of bile acids in PCD mice and patients.

Supplementary Tables include

Table S1: Characteristic information for donors, including healthy control, PCD patients and NonPCD.

Table S2: PCR primer sequences used in this study.

Table S3: Information of the reagents or kits used in the study.

Table S4: Concentration of 43 fecal bile acids metabolites among three humanized gut microbiota mice groups.

Table S5: The ratio of primary bile acids to secondary bile acids in feces of these grouped mice.

**Supplementary methods**

**Participants recruiting and sampling**

Post-cholecystectomy diarrhea (PCD) patients (n=10) were selected in Minhang hospital, Fudan university according to diarrheal criteria, ^1, 2^ as well as NonPCD patients (n=5) and healthy controls (HC, n=5). Clinical characters including age, sex, BMI, defecation frequency per day, stool output and fecal consistency by Bristol stool score (BSS) of these subjects were recorded and shown in Supplementary Table S1. Morning first stool samples were obtained from donors and suspended in an equal volume (w/v) PBS containing 20% glycerin, then frozen in liquid nitrogen immediately and preserved in -80 ℃ until use. Details of the inclusion criteria and exclusion criteria for those volunteers were shown as below.

Inclusion criteria and exclusion criteria for PCD and NonPCD patients:

1. Inclusion criteria: (1) underwent cholecystectomy in 2019; (2) within 18 years old to 65; (3) written informed consent; (4) PCD patients were selected when they met the diarrhea criteria -loose stool, increased defecation frequency (at least 3 times per day) or urgency, and significant altered fecal appearance graded by Bristol stool score. ^1, 2^

2. Exclusion criteria: (1) surgical histories of gastrointestinal tract; (2) medical history of irritable bowel syndrome (IBS), informatory bowel diseases (IBD), constipation and infective or idiopathic diarrhea; (3) medication history of antibiotics, probiotics or medicines known to affect gut microbiota; (4) history of chronic diseases.

Healthy controls (HC) were recruited by following criteria:

1. Inclusive criteria: (1) aging within 18 years old to 65; (2) written informed consent; (3) denying of gallbladder removal surgery or history of gastrointestinal surgery; (4) fecal consistency scores for 3 and 4 by BSS; (5) written informed consent.

2. Exclusive criteria: (1) chronic diseases including IBS, IBD, constipation and infective or idiopathic diarrhea; (2) administration of antibiotics, probiotics or medicines known to affect gut microbiota in recent 3 months.

**Extraction of microbial genome DNA and 16S rRNA amplicon sequencing**

According to manufacturers’ instructions,^3^ total genome DNA from human and mice fecal samples were extracted by DNA extraction kit. In the template of microbial DNA, Hypervariable regions (V3-V4) of 16S rRNA gene were amplified by using specific bacterial primers (338 F and 806 R) with the barcode, and processed to sequencing libraries through TruSeq® DNA PCR-Free Sample Preparation Kit. Sample amplicons were sequenced on an Illumina HiSeq platform (Illumina, MiSeq, USA) and 250 bp paired-end reads were generated.

**Bioinformatic processing on 16S rRNA data**

Alpha diversity is applied in analyzing complexity of gut microbiota diversity and were calculated with QIIME (Version 1.7.0). Beta diversity was used to evaluate differences among samples, which was analyzed by Bray-Curtis dissimilarity and the Jaccard similarity index. Bacterial community distance was estimated by the nonmetric multidimensional scaling (NMDS) assay, principal coordinate analysis (PCoA) and the unweighted pair‐group method with arithmetic means (UPGMA) analysis among samples.^4^ Moreover, FAPROTAX^5^ and Tax4Fun^6^ were employed to predict bacterial metagenome functions in metabolism and ecology, and differentially-abundant functions in KEGG pathway level 3 between NonPCD and PCD groups were ranked by unsupervised RandomForest classification.^4^

**Targeted bile acid metabolism in fecal samples**

Fecal bile acid metabolites from recipient mice including 43 individuals were analyzed by UPLC/MS as previously reported.^3^ The collected samples were dried in the lyophilizer. Then, 50 mg of each sample was mixed with 800 μL precooled methanol containing 20 ng/mL internal standards, vortexed for 1 min, and incubated at 4 °C for 30 min. After centrifugation at 12000 rpm for 10 min, the supernatants were diluted 100 times with methanol and quantified in multiple reaction monitoring (MRM) mode. The acquisition data was analyzed by MultiQuant software (AB sciex, USA) and the concentration of individual bile acid was calculated by comparing with internal standard.

Internal standard reagents: a panel of 43 bile acid metabolites were determined in feces of mice and patients, comprising glycolithocholic acid (GLCA), apocholic acid (apoCA), 7,12-diketolithocholic acid (7,12-diketoLCA), taurolithocholic acid (TLCA), isolithocholic acid (isoLCA), murideoxycholic acid (MDCA), 12-dehydrocholic acid (12-DHCA), tauro-β-muricholic acid (TβMCA), glycodehydrocholic acid (GDHCA), β-hyodeoxycholic acid (βHDCA), norcholic acid (NorCA), β-ursodeoxycholic acid (βUDCA), dehydrolithocholic acid (dehydroLCA), allolithocholic acid (alloLCA), ursocholic acid (UCA), allocholic acid (ACA), 23-nordeoxycholic acid (NorDCA), 3-dehydrocholic acid (3-DHCA), 6-ketolithocholic acid (6-ketoLCA), 7-ketodeoxycholic acid (7-ketoDCA), taurodeoxycholic acid (TDCA), hyodeoxycholic acid (HDCA), deoxycholic acid (DCA), taurochenodeoxycholic acid (TCDCA), taurohyodeoxycholic acid (THDCA), taurocholic acid (TCA), tauroursodeoxycholic acid (TUDCA), ursodeoxycholic acid (UDCA), chenodeoxycholic acid (CDCA), cholic acid (CA), glycoursodeoxycholic acid (GUDCA), glycohyodeoxycholic acid (GHDCA), glycochenodeoxycholic acid (GCDCA), glycodeoxycholicacid (GDCA), glycocholic acid (GCA), lithocholic acid (LCA), isodeoxycholic acid (isoDCA), 12-ketolithocholic acid (12-ketoLCA), ω-muricholic acid (ωMCA), taurohyocholic acid (THCA), λ-muricholic acid (λMCA), α-muricholic acid (αMCA), β-muricholic acid (βMCA).

**Targeted profiling fecal tryptophan metabolism**

A total of 100 ± 5mg fecal samples were placed into a 2 mL centrifuge tube after being thawed on ice, then, mixed with 10 μL internal standard solution and 0.5 mL water- acetonitrile-methanol (1:2:2, v/v/v) solution. After several ceramic beads were added, they were homogenated using Biospec MiniBeadbeater. After centrifugation at 12000g for 10 min, 400 μL supernatant was collected and dried under nitrogen gas. The residue was re-dissolved in 100 μL acetonitrile-water (1:1, v/v) and then centrifuged again. The supernatant was analyzed by HPLC-MS/MS and HPLC-MS/MS condition was described in supplementary methods. Integrative crosstalk among these tryptophan metabolites was disclosed by KEGG enrichment analysis and depicted in KEGG map.^7^ Total 5-HT excretion of each donor was calculated by the concentration (ng/g) × defecation output (g).

Internal standard reagents: a panel of 23 tryptophan metabolites were determined in feces of NonPCD and PCD patients, comprising kynurenate, tryptophan, indole-3-propionic acid (IPA), quinolinic acid, indoleacetate, serotonin, indole-3-acetaldehyde (IAAld), tryptamine, xanthurenate, indole-3-lactic acid (ILA), cinnavalininate, nicotinamide adenine dinucleotide (NAD), 5-hydroxyindole-3-acetic acid, N-formyl-kynurenine, picolinic acid, indole-3-carboxaldehyde, 5-hydroxy-L-tryptophan (5-HTP), L-kynurenine, melatonin, 3-hydroxyanthranilic acid, 3-indoxyl sulfate, 3-hydroxyl-L-kynurenine and indoxyl-b-D-glucuronide.

**HPLC-MS/MS condition**

The separation was performed on a UPLC system (Agilent 1290 Infinity UHPLC) on a C-18 column (Waters, CSH C18 1.7μm, 2.1 mm×100 mm column) by gradient elution. Eluent A was acetonitrile and Eluent B contained 20 mM ammonium formate buffer (pH 3.7). The gradient elution program was as follows: 0 min = 15% B, 2min =15% B, 9 min = 98% B, 11 min = 98% B and 11.5 min = 15% B, and 14 min=15% B. Before injecting the next sample, the column was equilibrated with the initial mobile phase for 5 min. The flow rate was constant at 0.4 mL/min and the column temperature was set at 50 ℃. The ESI positive source conditions were as follows: source temperature: 550℃; ion Source Gas1 (Gas1): 55; Ion Source Gas2 (Gas2): 55; Curtain gas (CUR): 40; ionSapary Voltage Floating (ISVF): +4500 V; The ESI negative source conditions were as follows: source temperature: 550℃; ion Source Gas1 (Gas1): 55; Ion Source Gas2 (Gas2): 55; Curtain gas (CUR): 40; ionSapary Voltage Floating (ISVF): -4500 V. The APCI source conditions were as follows: source temperature: 550℃; ion Source Gas1 (Gas1): 55; Ion Source Gas2 (Gas2): 55; Curtain gas (CUR): 40; ionSapary Voltage Floating (ISVF): +5500 V. QTRAP 5500 mass spectrometer (AB SCIEX) was performed in positive switch mode and MRM method was used for mass spectrometry quantitative data acquisition, and MultiQuant (v3.0.3) or Analyst (v1.6.2) was used for data processing.

**Reference**

1. Arasaradnam RP, Brown S, Forbes A, Fox MR, Hungin P, Kelman L, Major G, O'Connor M, Sanders DS, Sinha R, et al. Guidelines for the investigation of chronic diarrhoea in adults: British Society of Gastroenterology, 3rd edition. Gut. 2018; 67(8):1380-1399.doi:10.1136/gutjnl-2017-315909.
2. Smalley W, Falck-Ytter C, Carrasco-Labra A, Wani S, Lytvyn L, Falck-Ytter Y. AGA Clinical Practice Guidelines on the Laboratory Evaluation of Functional Diarrhea and Diarrhea-Predominant Irritable Bowel Syndrome in Adults (IBS-D). Gastroenterology. 2019; 157(3):851-854.doi:10.1053/j.gastro.2019.07.004.
3. Xu Y, Jing H, Wang J, Zhang S, Chang Q, Li Z, Wu X, Zhang Z. Disordered Gut Microbiota Correlates With Altered Fecal Bile Acid Metabolism and Post-cholecystectomy Diarrhea. Front Microbiol. 2022; 13.doi:10.3389/fmicb.2022.800604.
4. Zhang SL, Mao YQ, Zhang ZY, Li ZM, Kong CY, Chen HL, Cai PR, Han B, Ye T, Wang LS. Pectin supplement significantly enhanced the anti-PD-1 efficacy in tumor-bearing mice humanized with gut microbiota from patients with colorectal cancer. Theranostics. 2021; 11(9):4155-4170.doi:10.7150/thno.54476.
5. Xu, Z. Xu, W. Zhang, L. Ma, Y. Li, Y. Li, G. Nghiem, L. D. Luo, W. et al. Bacterial dynamics and functions driven by bulking agents to mitigate gaseous emissions in kitchen waste composting. Bioresour Technol. 2021;332:125028. doi: 10.1016/j.biortech.2021.125028.
6. Sun S, Jones RB, Fodor AA. Inference-based accuracy of metagenome prediction tools varies across sample types and functional categories. Microbiome. 2020;8(1):46. doi: 10.1186/s40168-020-00815-y.
7. Liu, X. Han, M. Zhao, C. Chang, C. Zhu, Y. Ge, C. Yin, R. Zhan, Y. Li, C. Yu, M. et al. KeggExp: a web server for visual integration of KEGG pathways and expression profile data. Bioinformatics. 2019;35(8):1430-1432. doi: 10.1093/bioinformatics/bty798.

**Supplementary Figures:**


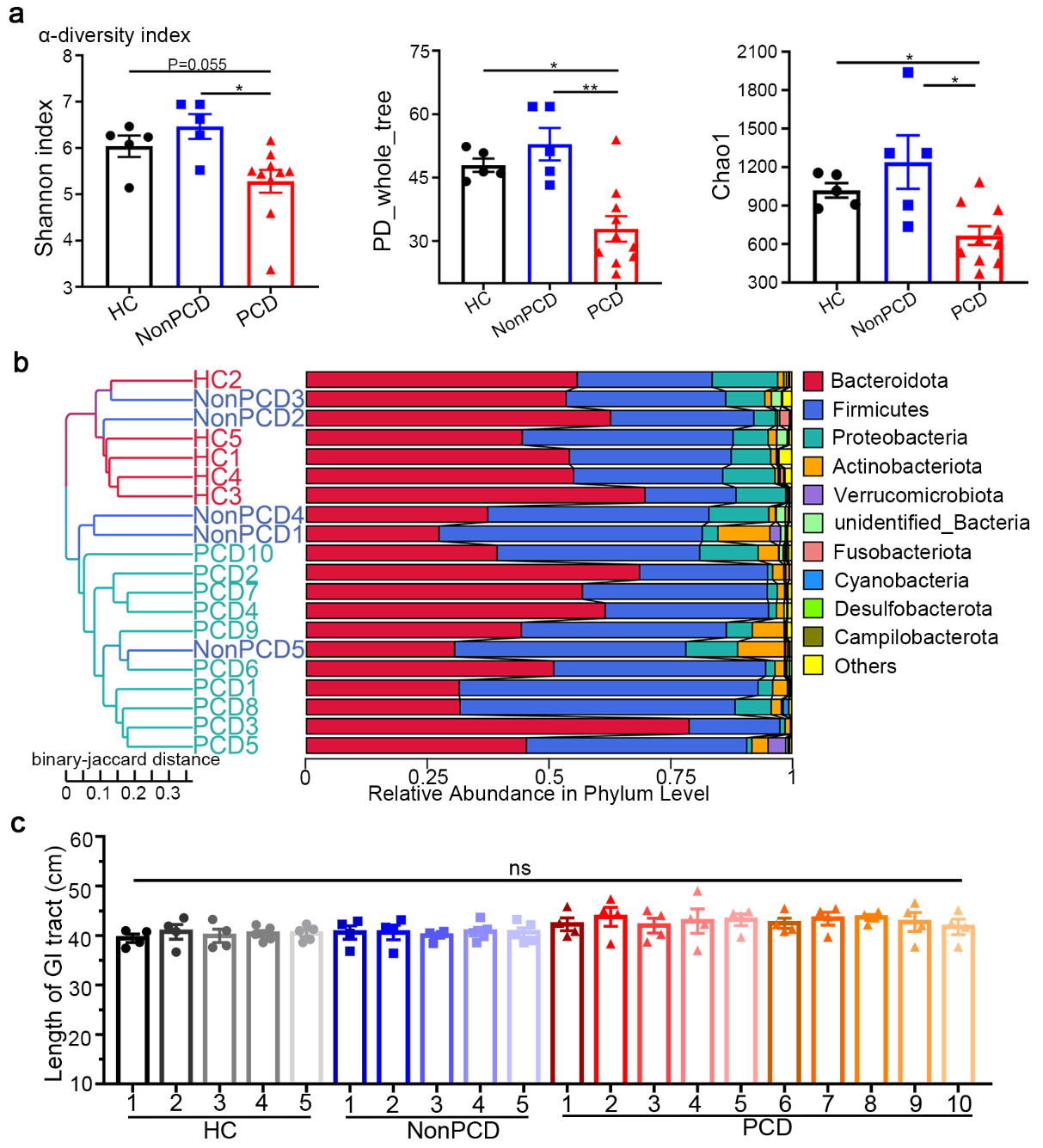


**Figure S1**: Reduced bacterial diversity and changed composition in feces from PCD donors. (a) attenuated gut microbial richness and evenness by decreased α-diversity (Shannon index, PD-whole-tree and Chao1) in healthy control (HC, n=5), NonPCD (n=5) and PCD (n=10) individuals. (b) Disordered fecal bacterial structure by unweighted pair-group method with arithmetic means (UPGMA) tree based on bray-curtis distance (left panel) and bacterial top 10 abundance and structure in phylum of each donor among three groups (right panel). (c) Total length of whole gastrointestinal (GI) tract of mice transplanted with donors’ fecal microbiota (n=4). Data are shown as mean ± SEM; *p<0.05, ** p<0.01, *** p<0.005, ns for not significantly.


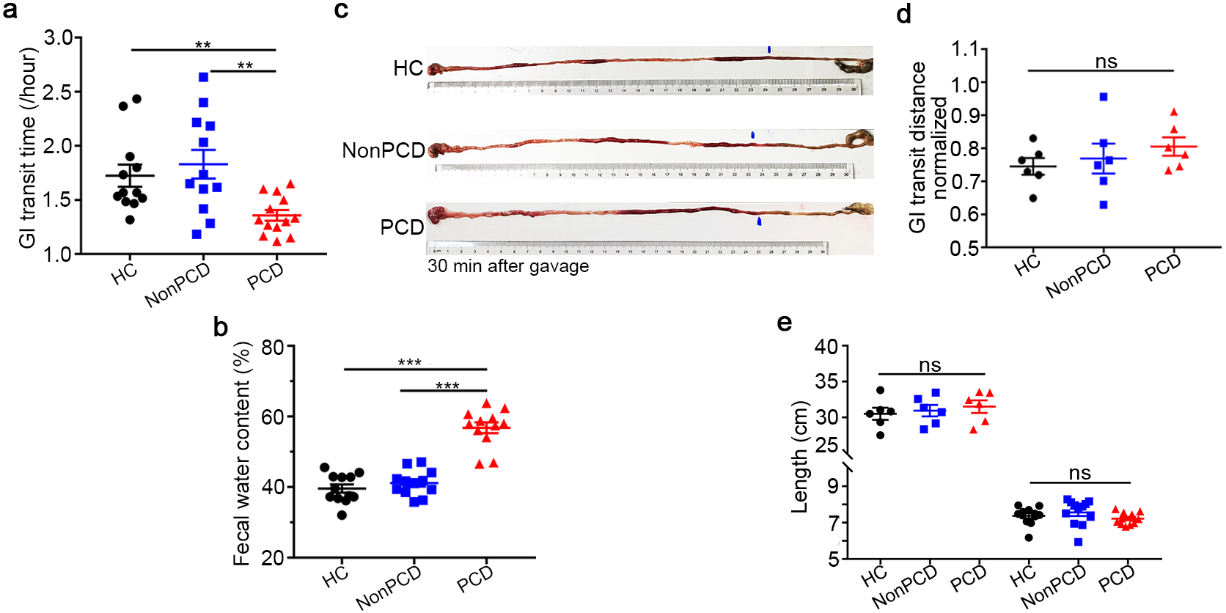


**Figure S2**: Compatible peristaltic motility of small intestine among three humanized gut microbiota mice groups. (a) Whole GITT in mice humanized with gut microbiota mixture from 5 HC donors, 5 NonPCD and 5 PCD patients, n=12/group. (b) Fecal water content in three groups (n=12). (c) Photographs for peristalsis distance of carmine solution in small intestine of each group 30 min after the marker solution gavage. (d) Gastrointestinal transit distance in small intestine were compatible among three groups (n=6), normalized by the length of whole small intestine. (e) Length of small intestine and colon among three groups. Data are expressed in mean ± SEM; * p<0.05, ** p<0.01, *** p<0.005, ns for not significant.


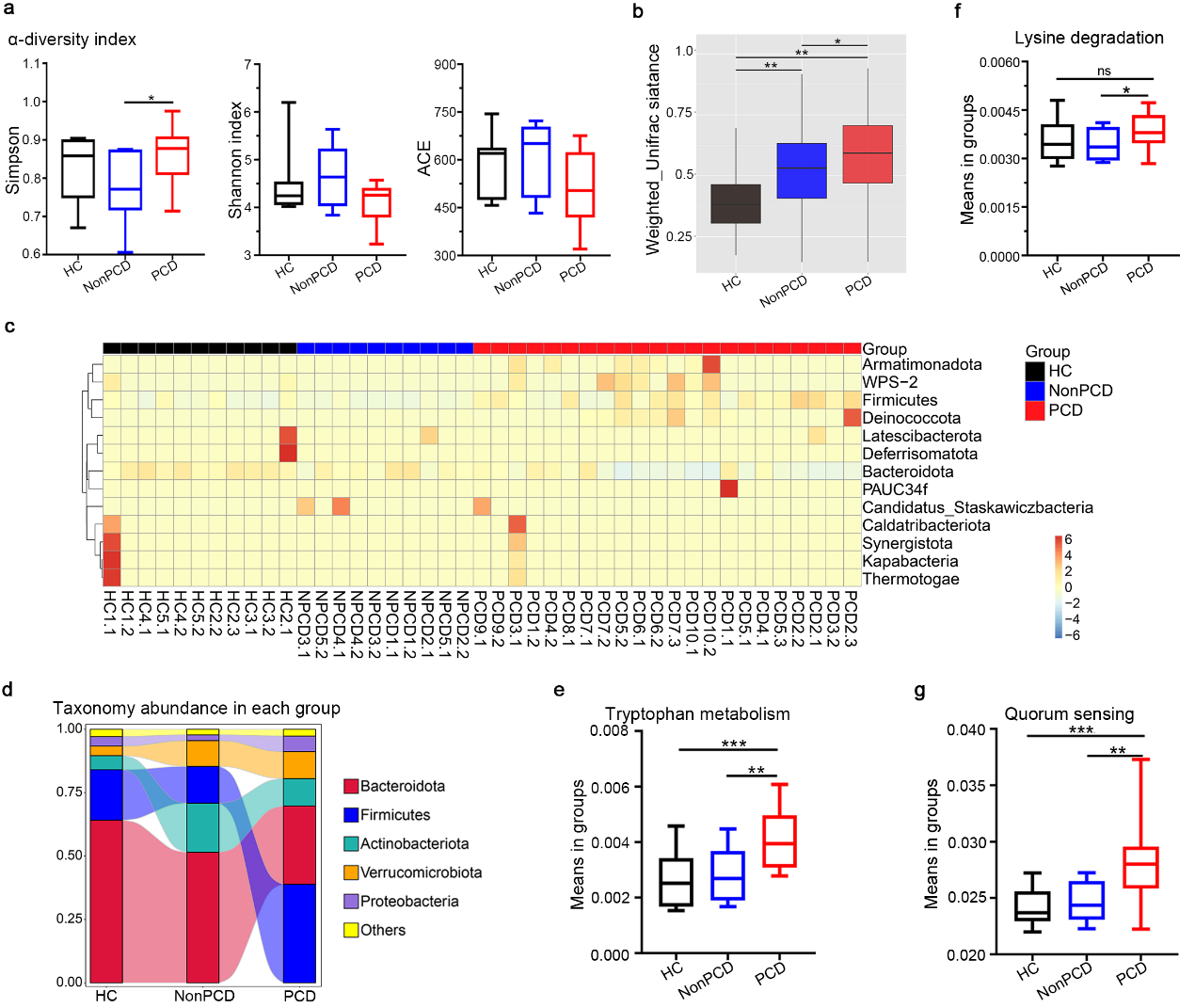


**Figure S3**: Declined gut microbial diversity and altered bacterial structure in recipient mice. (a) Decreased bacterial richness and evenness by reduced α-diversity (Simpson, Shannon index and ACE) in humanized mice, n=11 for HC, n=10 for NonPCD and n=22 for PCD group. (b) Beta-diversity index by weighted Unifrac distance (Wilcox rank sum test) in three groups. (c) Heatmap showing changed bacterial structure (especially *Firmicutes* and *Bacteroidota*) in phylum among three groups. (d) Sankey diagram showing microbial differences in phylum level among three groups. (e, f, g) Bacterial function predicted by Tax4Fun based on KEGG pathway analysis among three groups, lysine degradation in (e), tryptophan metabolism in (f) and quorum sensing in (g). Data are expressed as mean ± SEM. * p<0.05, ** p<0.01, *** p<0.005.


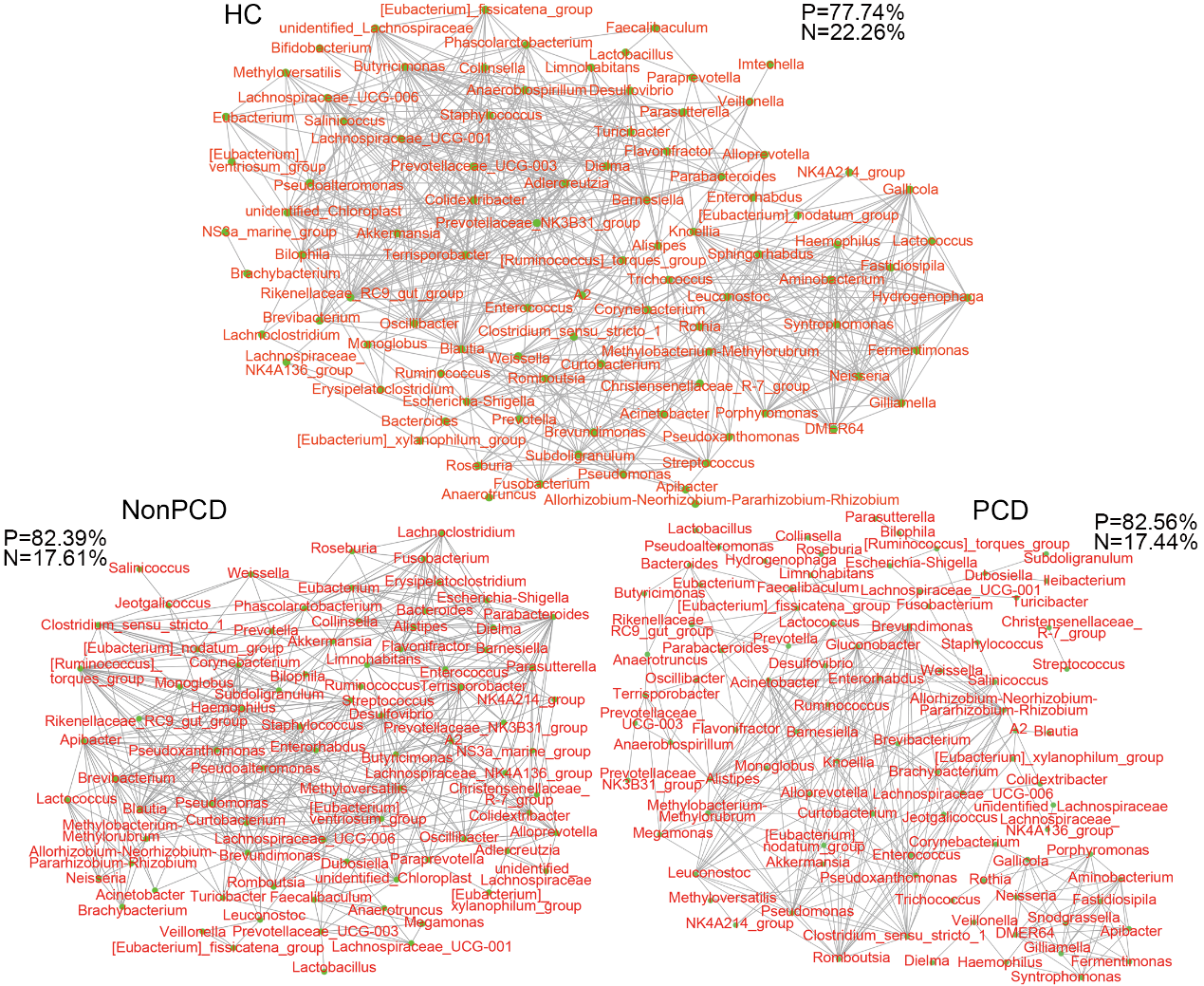


**Figure S4**: Reduced co-occurrence network among top 100 genera in PCD mice. Representative co-abundance network showing inter-relationship among genus bacteria in three groups, respectively. Correlations among top 100 genera were analyzed by the abundance of each genus microbe, and the correlation coefficients (P_fdr_<0.05,|r|>0.7) were considered as clear links and visualized by Cytoscape v3.8.2. Multiplicity testing were conducted and P value was adjusted using the methods of Benjamini, Hochberg, and Yekutieli to control the false discovery rate (FDR). P_fdr_<0.05 was considered significant. Genera were presented in green dots labeled with red and mutual correlation in gray line in three groups. There existed declined co-relationship network in PCD group with only 281 correlations, but 638 in HC and 494 in NonPCD groups. P, positive correlation; N, negative correlation.


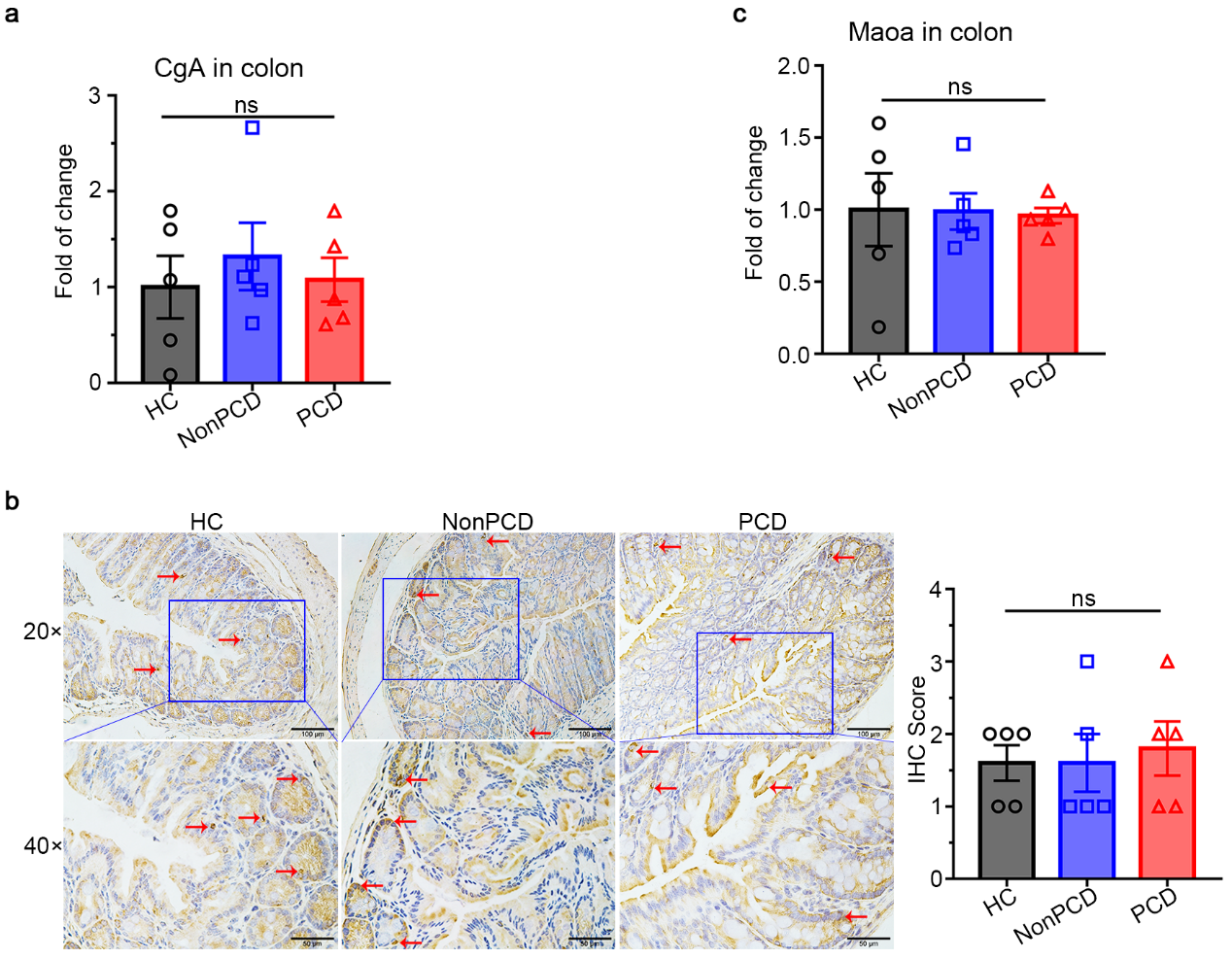


**Figure S5**: Undifferentiated colonic enterochromaffin (ECs) cells and compatible Maoa among three groups. (a) Parallel expression of chromogranin A+ (CgA) indicating insignificant proliferation of colonic ECs in mice, n=5/group. (b) Representative IHC photographs of strained CgA in colon (left panel) and the IHC score (right panel), n=5/group. (c) Relative expression of Maoa for 5-HT degradation in three grouped mice (n=5). Data are expressed in mean ± SEM; * p<0.05, ** p<0.01, ns for not significant.


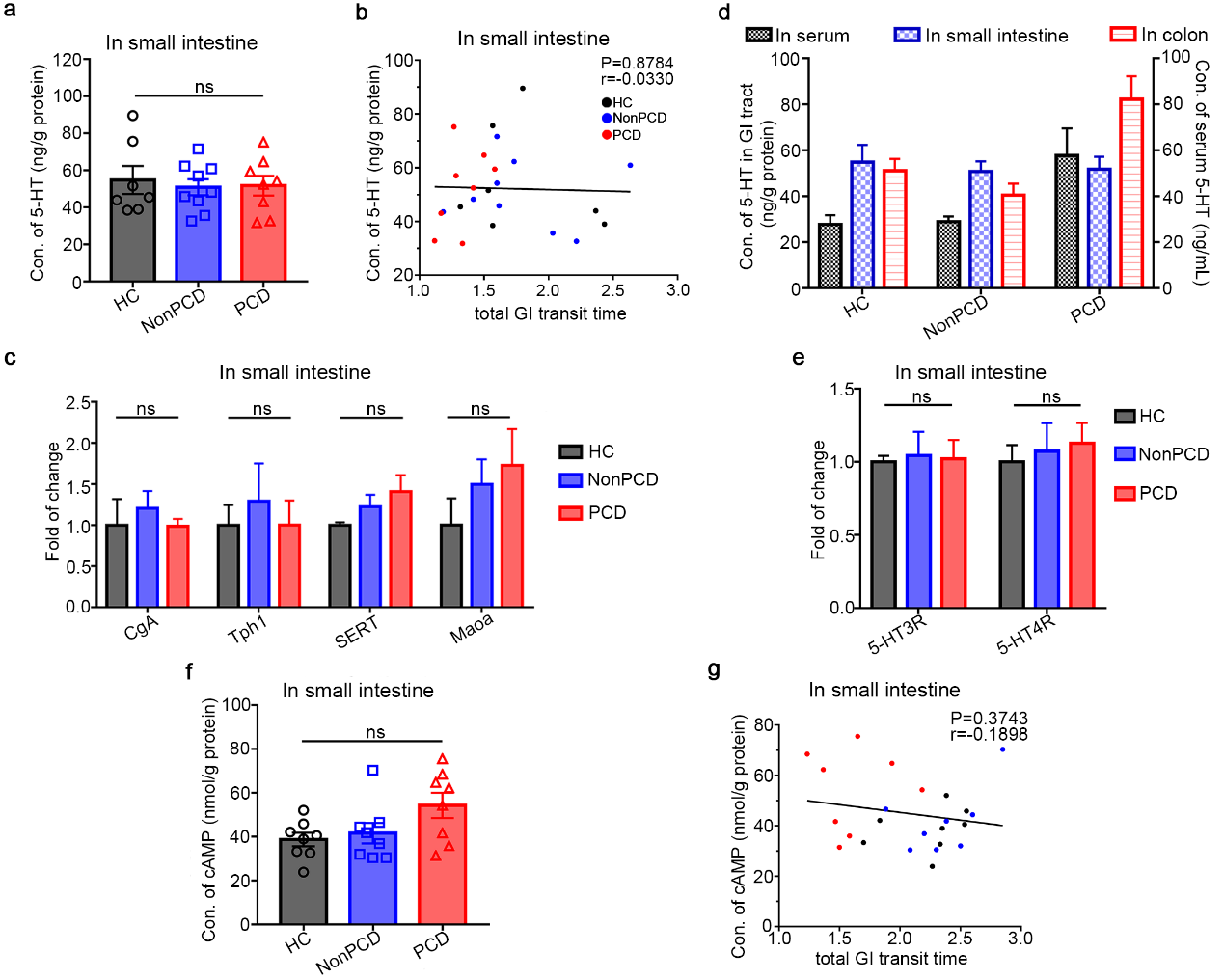


**Figure S6**: Invariant 5-HT level with unchanged and inactivated 5-HT receptors in small intestine among three grouped mice. (a) Level of 5-HT in small intestine, normalized by total protein in mice, n=7-9. (b) Insignificant relation between 5-HT level in small intestine and total GI transit time. (c) Serotonin level in serum, small intestine, and colon among three grouped mice. (d) Relative expressions of vital intermediates relative to *β-actin* for 5-HT metabolism in small intestine of mice among groups (n=3-4). (e) Negative correlation with significance between colonic cAMP and total GI transit time. (f) Relative expressions of 5-HT3R and 5-HT4R in small intestine among three grouped mice, n=5. (g, h) Compatible level of cAMP among three grouped mice (g) and its insignificant correlation with total gastrointestinal transit time (h) indicating inactivated 5-HTR in small intestine, n=8/group. Data are expressed in mean ± SEM; ns for not significant.


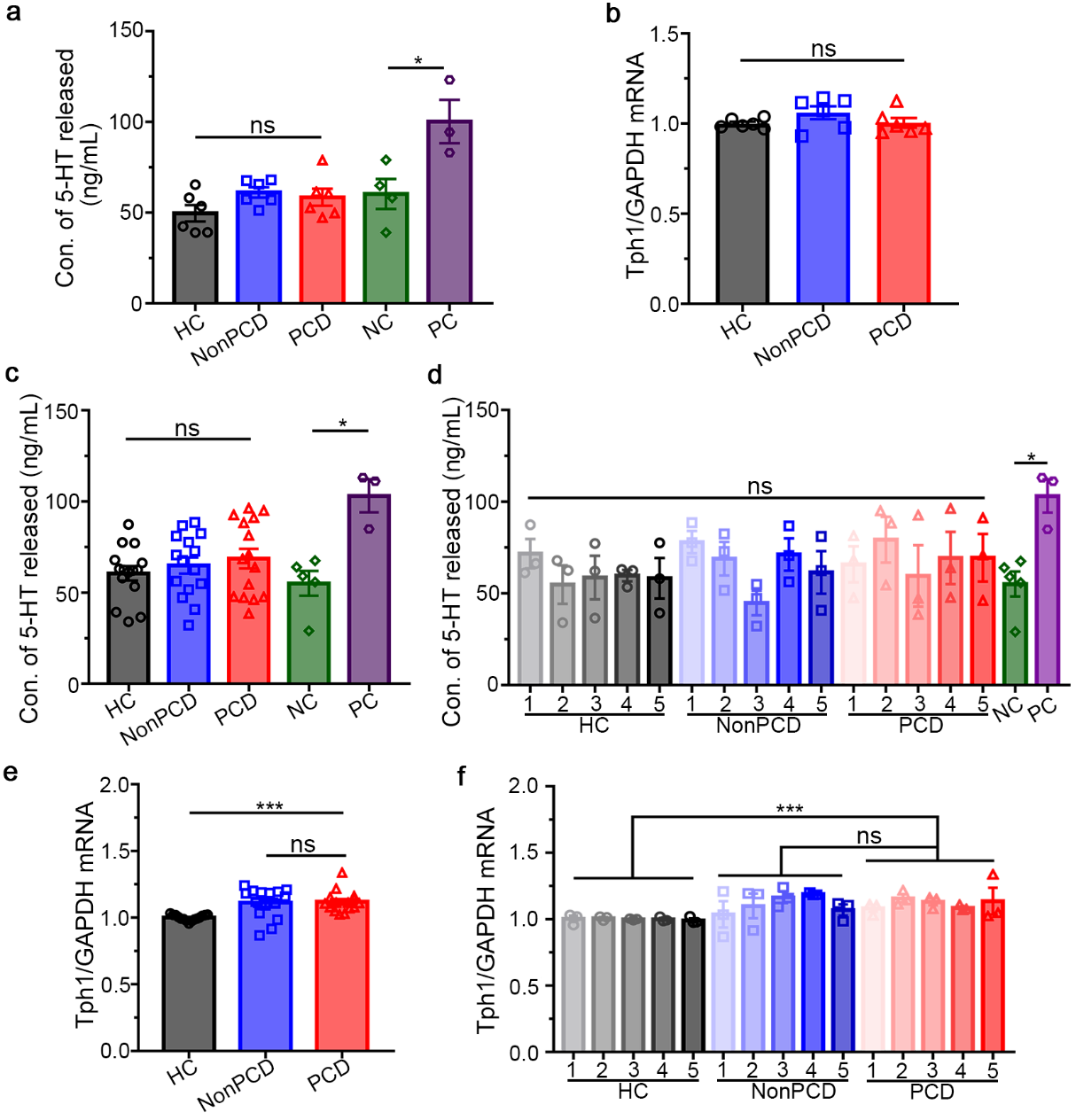


**Figure S7**: Bacteria from PCD mice and patients failed to elevated 5-HT level and stimulate Tph1 in RIN14B cells. (a) Compatible 5-HT level in RIN14B cell supernatants after exposure to washed bacteria from recipient mice, normal 5-HT level was determined as normal control (NC) and 15 μM ionomycin was added as positive control (PC), n=3-6. (b) Relative expression of intracellular Tph1 to GAPDH in RIN14B cells when exposed to bacteria from recipient mice, n=6. (c, d) Level of 5-HT in RIN14B cell supernatants when exposed to grouped patients’ fecal bacteria (c) and 15 individuals (d), n=3/donor. (e, f) Indiscrimination of Tph1 expressions in three grouped patients (e) and among 15 individuals (f), n=3/donor.


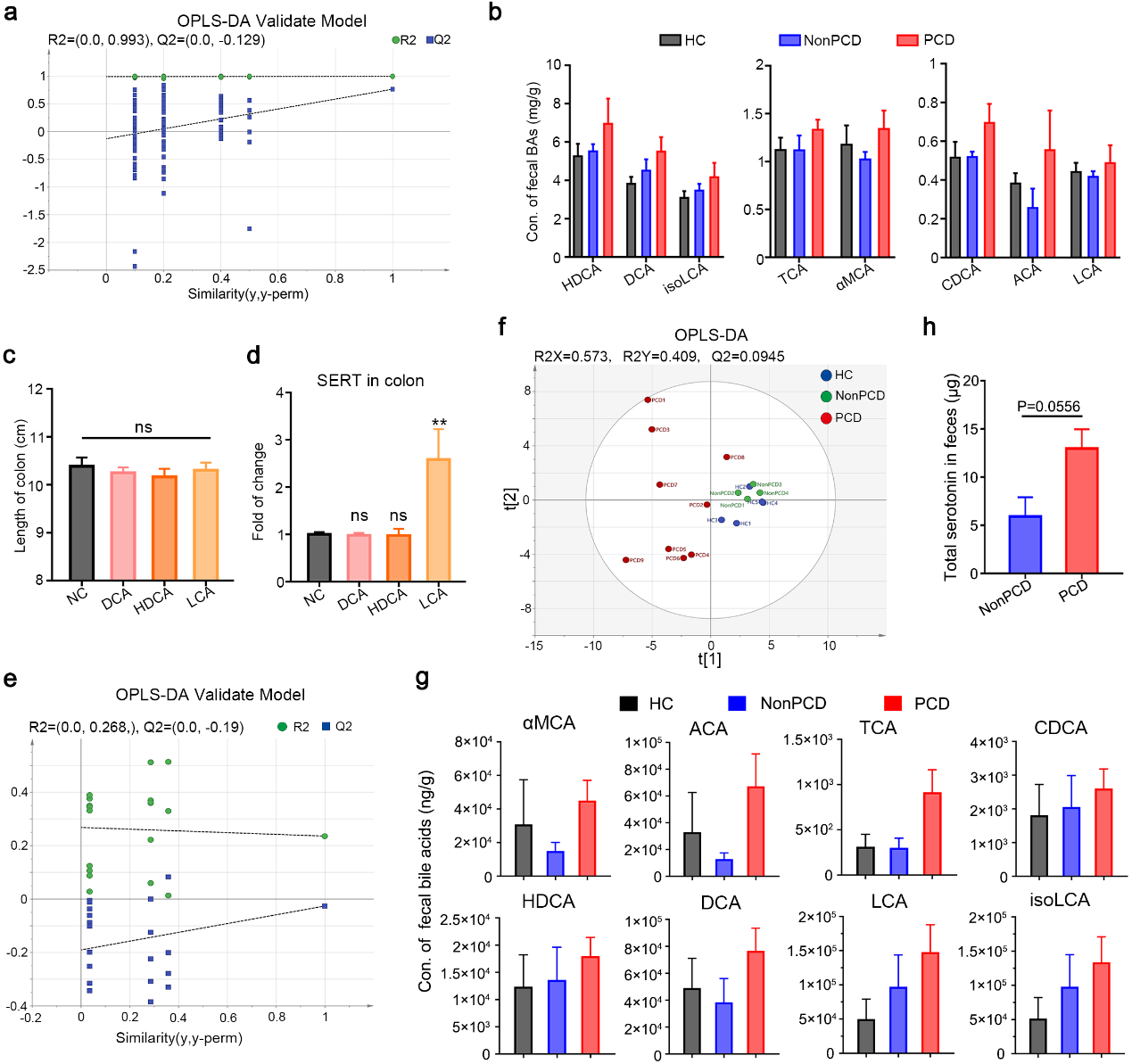


**Figure S8**: Overabundant BAs metabolites in feces of PCD patients. (a) OPLS-DA validate model of fecal bile acid metabolism in three grouped mice by permutations plot. (b) Quantified data showing increasing fecal BAs metabolites in PCD mice. (c) Length of colon with no significance among bile acids treated mice and control mice, n=9-12. (d) Elevated expression of SERT to β-actin by LCA, but not DCA and HDCA, n=5. (e) OPLS-DA validate model of fecal bile acid metabolism in three grouped patients by permutations plot. (f) OPLS-DA model showing the difference fecal BAs metabolism in PCD patients (n=9) from NonPCD (n=4) and HC (n=5). (g) Metabolic characteristics of specific bile acids metabolites in feces of clinical donors. (h) Increased total serotonin excretion in feces of PCD patients, compared to NonPCD patients, total serotonin excretion=fecal 5-HT concentration (ng/g)×fecal output (g), n=5. **P<0.01, ns for not significant.


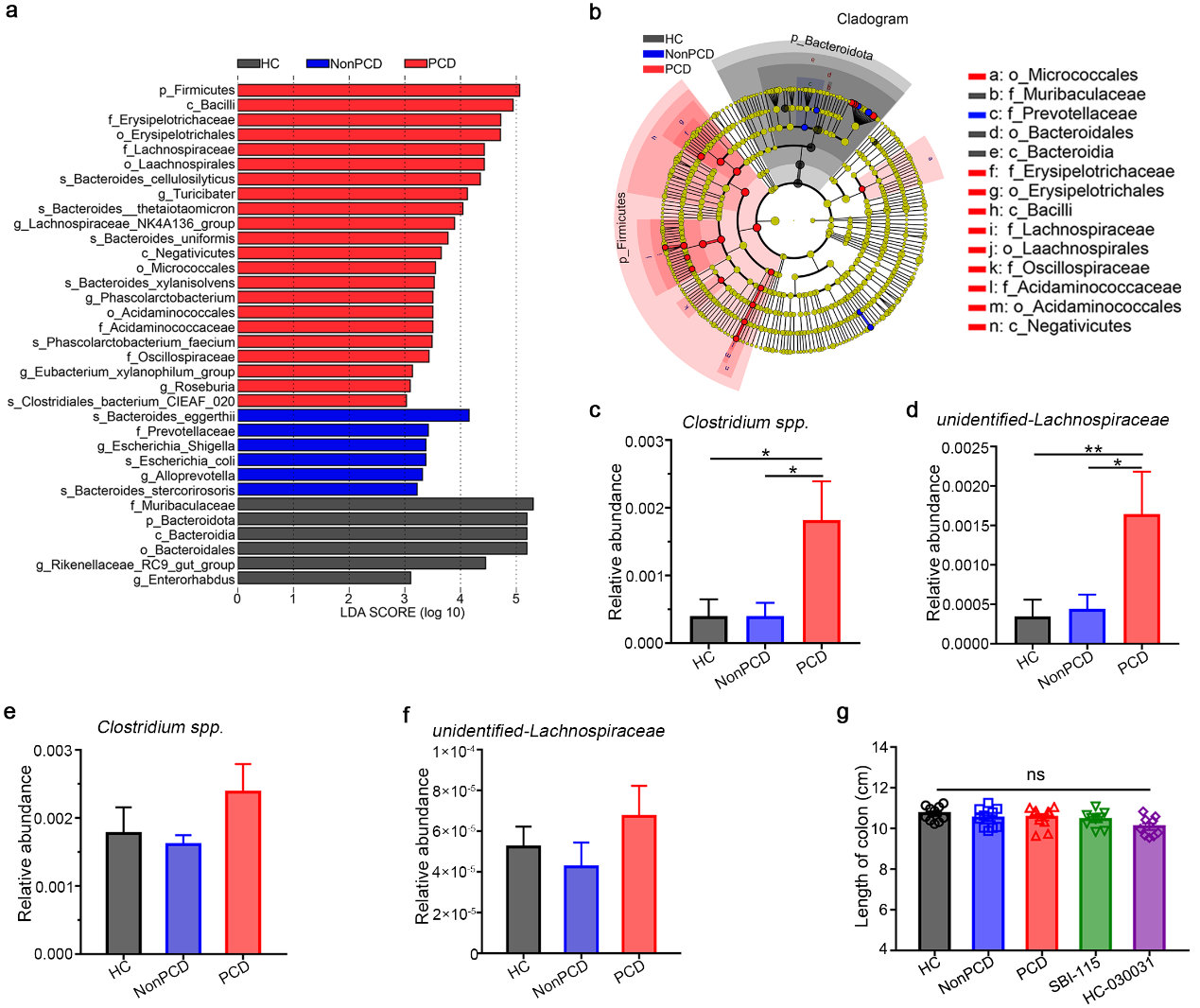


**Figure S9**: Overabundant fecal bacteria with metabolic capability of bile acids in PCD mice and patients. (a) Linear discriminant analysis (LDA) effect size (LEfSe) plot showing differential abundance of bacteria among three grouped mice (bacteria whose LDA score>3.0 were plotted). (b) Cladogram displaying specific fecal microbes abundant in three grouped mice. Yellow nodes show taxa with no significance, circle rings from inside to outside show taxonomic hierarchy from phylum to species, respectively. (c, d) Increased relative abundance of fecal bacteria encoding 7-dehydroxylases including *Clostridium spp.* (c) and *unidentified-Lachnospiraceae* (d) in feces of PCD mice, n=10-22. (e, f) Increased relative abundance of *Clostridium spp.* (e) and *unidentified-Lachnospiraceae* (f) in feces of PCD patients, n=22-25. (g) Elevated fecal excretion of 5-HT in PCD patients, compared to NonPCD patients, total 5-HT excretion of each donor was calculated by the concentration (ng/g) × defecation output (g). (h) The length of colon from 5 grouped mice, n=9-11. Data are expressed in mean ± SEM; * p<0.05, ** p<0.01, ns for not significant.

**Supplementary Tables**

**Table S1**: Characteristic information for donors, including healthy control (HC), PCD

patients and NonPCD.

M, male; F, female; BSS, Bristol stool score.

| Donor | Sex | Age | BMI (kg/m^2^) | Defecation frequency (/day) | BSS | Fecal output (g/day) | Months after LC |
| --- | --- | --- | --- | --- | --- | --- | --- |
| HC1 | M | 32 | 21.37 | 1 | 4 | 105 |  |
| HC2 | M | 25 | 22.45 | 1 | 3 | 110 |  |
| HC3 | F | 28 | 19.29 | 1.5 | 3 | 142.5 |  |
| HC4 | F | 31 | 25.40 | 1 | 3 | 108 |  |
| HC5 | F | 33 | 22.86 | 1 | 3 | 92 |  |
| NonPCD1 | F | 41 | 25.71 | 1.5 | 2 | 195 | 12 |
| NonPCD2 | F | 22 | 21.48 | 1 | 2 | 110 | 11.5 |
| NonPCD3 | M | 46 | 22.88 | 1 | 3 | 70 | 16 |
| NonPCD4 | F | 38 | 24.03 | 1 | 3 | 59 | 11 |
| NonPCD5 | M | 52 | 20.05 | 1.5 | 3 | 115.5 | 15.5 |
| PCD1 | F | 57 | 28.04 | 6.5 | 7 | 370.5 | 13 |
| PCD2 | F | 38 | 27.76 | 2.5 | 7 | 400 | 12.5 |
| PCD3 | F | 53 | 21.5 | 3.5 | 6 | 420 | 12 |
| PCD4 | M | 34 | 27.47 | 2.5 | 6 | 512.5 | 12.5 |
| PCD5 | M | 37 | 23.93 | 3.5 | 6 | 385 | 8 |
| PCD6 | M | 36 | 22.86 | 3.5 | 6 | 661.5 | 12.5 |
| PCD7 | M | 35 | 28.37 | 4.5 | 5 | 387 | 11.5 |
| PCD8 | M | 52 | 26.35 | 3 | 6 | 330 | 14 |
| PCD9 | F | 42 | 21.22 | 3 | 5 | 195 | 15 |
| PCD10 | F | 32 | 23.88 | 2.5 | 7 | 280 | 12 |

**Table S2**: PCR primer sequences used in this study.

M for mouse, r for rat.

|  | Forward (5’-3’) | Reverse (5’-3’) |
| --- | --- | --- |
| Total Bacteria | ACTCCTACGGGAGGCAGCAG | GGACTACHVGGGTWTCTAAT |
| mTph1 | ACCATGATTGAAGACAACAAGGAG | TCAACTGTTCTCGGCTGATG |
| mCgA | ACTTCCATGCAGGCTACAAAGC | CTCTGTCTTTCCATCTCCATCCA |
| mSERT | CGCAGTTCCCAGTACAAGC | CGTGAAGGAGGAGATGAGG |
| mMaoa | GGAGAAGCCCAGTATCACAGG | GAACCAAGACATTAATTTTGTATTCTGAC |
| m5-HT3R | CCAGTCCTGACTGGCTGAG | AAGTCCTGAGGTCCTCCAAC |
| m5-HT4R | TAATGTTGGGAGGCTGCTGG | TGCAGACACAGATGGGGATG |
| mTGR5 | TCCTGTCAGTCTTGGCCTATGA | GGTGCTGCCCAATGAGATG |
| mTRPA1 | GGAGCAGACATCAACAGCAC | GCAGGGGCGACTTCTTATC |
| mβ-actin | CAGCTTCTTTGCAGCTCCTT | CTTCTCCATGTCGTCCCAGT |
| rTph1 | AACAAAGACCATTCCTCCGAAAG | TGTAACAGGCTCACATGATTCTC |
| rGAPDH | GCATCTTCTTGTGCAGTGCC | GATGGTGATGGGTTTCCCGT |

**Table S3**: Information of the reagents or kits used in the study.

| Reagents/Kits | Catalogue No. | Manufacturers |  |
| --- | --- | --- | --- |
| vancomycin | V871983 | Macklin |  |
| neomycin | N814740 | Macklin |  |
| metronidazole | M813525 | Macklin |  |
| amphotericin-B | A800428 | Macklin |  |
| ampicillin | A800429 | Macklin |  |
| carmine red | C8540 | Solarbio |  |
| methylcellulose | M8070 | Solarbio |  |
| DNA extraction kit | DP303-02 | TIANGEN |  |
| TruSeq® DNA PCR-Free Kit | 20015963 | Illumina |  |
| RNA-Quick Purification Kit | RN001/RN002 | ES Science |  |
| Prime Script™ RT Master Mix | RR036A | TaKaRa, Bio |  |
| PowerUp™ SYBR™ Green Master Mix | A25742 | Thermo Fisher Scientific |  |
| 5-HT ELISA Kit |  | MultiScience |  |
| Tryptamine ELISA Kit |  | MultiScience |  |
| cAMP ELISA Kit |  | MultiScience |  |
| Chromogranin A rabbit polyclonal antibody | 10529-1-AP | Proteintech |  |
| HTR3A rabbit antibody | #32946 | Signalway Antibody |  |
| HTR4 rabbit polyclonal antibody | 10529-1-AP | Proteintech |  |
| Anti-GPCR TGR5 antibody | Ab72608 | Abcam |  |
| TRPA1 Polyclonal antibody | 19124-1-AP | Proteintech |  |
| Bile acid standards |  | yuanye Bio-Tech |  |
| Fetal bovine serum | 10099141C | Gbico^TM^ |  |
| Fluoxetine |  | Macklin |  |
| tryptophan metabolites standards |  | Sigma-Aldrich/Steraloids |  |
| Isolithocholic acid | GC47466 | Glpbio Technology |  |
| Lithocholic acid | 434-13-9 | MedChemExpress |  |
| Allocholic acid | B22337 | yuanye Bio-Tech |  |
| Chenodeoxycholic acid | B20347 | yuanye Bio-Tech |  |
| Hyodeoxycholic acid | 83-49-8 | Sigma |  |
| Deoxycholic acid | 83-44-3 | Sigma |  |
| α-Muricholic acid | S22142 | yuanye Bio-Tech |  |
| Taurocholic acid | B26949 | yuanye Bio-Tech |  |
| Ionomycin | S48414 | yuanye Bio-Tech. |  |
| Alosetron | 122852-69-1 | Glpbio Technology |  |
| GR113808 | 144625-51-4 | Abcam | |
| LX-1606 Hippurate | 1033805-22-9 | Liqi biomedical research center | |
| SBI-115 | 882366-16-7 | yuanye Bio-Tech | |
| HC-030031 | 349085-38-7 | TargetMol and Cayman Chemical | |

**Table S4**: Concentration of 43 fecal bile acids metabolites (ng/g) among three humanized gut microbiota mice groups.

| Sample Name | HC1 | HC2 | HC3 | HC4 | HC5 | NP1 | NP2 | NP3 | NP4 | NP5 | PCD1 | PCD2 | PCD 3 | PCD4 | PCD5 |
| --- | --- | --- | --- | --- | --- | --- | --- | --- | --- | --- | --- | --- | --- | --- | --- |
| TDCA | 206 | 105 | 163 | 87 | 138 | 70 | 171 | 108 | 115 | 121 | 109 | 187 | 198 | 164 | 117 |
| HDCA | 7406 | 5256 | 5399 | 3350 | 4848 | 4310 | 5290 | 6407 | 5315 | 6229 | 5527 | 2477 | 9648 | 8118 | 8950 |
| GUDCA | 5 | 2 | 0 | 3 | 3 | 2 | 1 | 2 | 2 | 3 | 3 | 2 | 4 | 3 | 2 |
| GHDCA | 20 | 15 | 23 | 11 | 23 | 6 | 4 | 38 | 17 | 7 | 16 | 8 | 7 | 11 | 16 |
| DCA | 3321 | 2654 | 4277 | 4689 | 4140 | 4961 | 4201 | 4427 | 2652 | 6294 | 3838 | 4372 | 8219 | 5773 | 5259 |
| TCDCA | 311 | 148 | 219 | 134 | 221 | 99 | 221 | 164 | 168 | 180 | 191 | 285 | 182 | 238 | 176 |
| THDCA | 345 | 235 | 76 | 22 | 39 | 22 | 14 | 17 | 67 | 41 | 15 | 10 | 32 | 83 | 20 |
| GCDCA | 8 | 5 | 8 | 8 | 7 | 4 | 4 | 5 | 7 | 6 | 6 | 7 | 8 | 6 | 6 |
| TCA | 1550 | 787 | 1224 | 978 | 1067 | 702 | 1622 | 1043 | 969 | 1255 | 1468 | 1671 | 1080 | 1232 | 1213 |
| GDCA | 9 | 6 | 9 | 10 | 6 | 3 | 4 | 8 | 6 | 6 | 7 | 7 | 7 | 5 | 5 |
| TUDCA | 518 | 285 | 814 | 380 | 192 | 443 | 350 | 315 | 190 | 804 | 170 | 467 | 600 | 202 | 294 |
| UDCA | 2 | 4 | 5 | 3 | 6 | 3 | 4 | 1 | 1 | 16 | 1 | 3 | 7 | 3 | 12 |
| CDCA | 605 | 649 | 195 | 591 | 536 | 478 | 615 | 541 | 470 | 490 | 539 | 391 | 862 | 890 | 793 |
| GCA | 22 | 8 | 19 | 37 | 29 | 10 | 12 | 51 | 20 | 28 | 32 | 14 | 14 | 18 | 13 |
| CA | 4761 | 1838 | 5109 | 8683 | 5170 | 3845 | 2825 | 5580 | 2845 | 10073 | 5120 | 3650 | 4221 | 4672 | 3321 |
| GLCA | 9 | 6 | 13 | 4 | 3 | 1 | 1 | 3 | 1 | 0 | 1 | 14 | 3 | 2 | 1 |
| apoCA | 2326 | 1968 | 1523 | 1454 | 1912 | 636 | 1055 | 942 | 1340 | 2187 | 1538 | 1230 | 1002 | 1014 | 1336 |
| 7,12-diketoLCA | 3 | 2 | 5 | 3 | 6 | 2 | 1 | 6 | 2 | 3 | 7 | 5 | 10 | 10 | 1 |
| TLCA | 92 | 30 | 25 | 14 | 11 | 16 | 21 | 24 | 29 | 22 | 5 | 24 | 32 | 26 | 20 |
| alloLCA | 4095 | 2624 | 4130 | 2986 | 3025 | 3928 | 4993 | 3922 | 3233 | 2554 | 2765 | 2481 | 6368 | 5095 | 6261 |
| LCA | 496 | 484 | 562 | 300 | 363 | 440 | 474 | 350 | 472 | 346 | 288 | 253 | 558 | 623 | 714 |
| MDCA | 6376 | 5308 | 3655 | 2983 | 4122 | 3763 | 4617 | 5482 | 5060 | 5376 | 4389 | 2386 | 6773 | 7360 | 5883 |
| 12-DHCA | 2 | 2 | 2 | 0 | 3 | 11 | 3 | 12 | 8 | 12 | 7 | 5 | 14 | 1 | 8 |
| TβMCA | 1585 | 788 | 1179 | 946 | 1124 | 646 | 1262 | 752 | 733 | 1030 | 1311 | 1774 | 971 | 943 | 919 |
| GDHCA | 1 | 10 | 10 | 9 | 69 | 11 | 16 | 2 | 4 | 9 | 2 | 1 | 12 | 10 | 6 |
| βMCA | 3252 | 1528 | 8390 | 9823 | 9747 | 5337 | 6095 | 3423 | 2810 | 6389 | 8623 | 4056 | 7701 | 4048 | 8300 |
| βHDCA | 2 | 21 | 16 | 19 | 59 | 56 | 22 | 20 | 29 | 38 | 39 | 149 | 29 | 33 | 20 |
| NorCA | 188 | 105 | 267 | 158 | 164 | 142 | 68 | 232 | 254 | 247 | 152 | 197 | 235 | 223 | 207 |
| βUDCA | 5032 | 4576 | 3592 | 3688 | 5180 | 3616 | 4214 | 5157 | 3015 | 5345 | 5487 | 3164 | 6331 | 4354 | 5445 |
| dehydroLCA | 1584 | 1074 | 1520 | 954 | 1298 | 622 | 892 | 758 | 1360 | 1059 | 1271 | 992 | 1018 | 730 | 1249 |
| isoLCA | 3679 | 2004 | 3993 | 2760 | 3020 | 3738 | 4553 | 3589 | 2844 | 2594 | 2605 | 2361 | 6218 | 4243 | 5379 |
| UCA | 5838 | 1937 | 5126 | 10134 | 5119 | 3744 | 2744 | 7542 | 3224 | 11443 | 5056 | 3670 | 4033 | 6215 | 3342 |
| ACA | 444 | 337 | 555 | 246 | 329 | 55 | 99 | 146 | 551 | 431 | 220 | 1211 | 800 | 72 | 464 |
| NorDCA | 46 | 29 | 7 | 28 | 13 | 11 | 18 | 17 | 22 | 46 | 6 | 3 | 31 | 4 | 12 |
| 3-DHCA | 6 | 1 | 19 | 37 | 14 | 30 | 67 | 81 | 6 | 22 | 23 | 8 | 11 | 3 | 14 |
| 6-ketoLCA | 2533 | 2311 | 1526 | 1544 | 1930 | 632 | 1022 | 914 | 1363 | 2272 | 1574 | 1256 | 1011 | 937 | 1359 |
| 7-ketoDCA | 2 | 41 | 22 | 7 | 42 | 11 | 4 | 10 | 7 | 11 | 4 | 2 | 1 | 4 | 7 |
| 12-ketoLCA | 0 | 1 | 0 | 0 | 1 | 0 | 0 | 0 | 0 | 0 | 0 | 2 | 4 | 0 | 0 |
| THCA | 1407 | 774 | 971 | 905 | 1056 | 643 | 1574 | 931 | 1000 | 1146 | 1214 | 1628 | 980 | 1036 | 1043 |
| isoDCA | 10 | 1 | 4 | 8 | 9 | 10 | 2 | 5 | 12 | 24 | 3 | 2 | 1 | 1 | 6 |
| λMCA | 1613 | 3568 | 1654 | 18785 | 11821 | 16912 | 13649 | 15932 | 10429 | 29997 | 9696 | 5384 | 27458 | 7429 | 9808 |
| αMCA | 1789 | 1233 | 1282 | 542 | 1032 | 999 | 1128 | 750 | 1217 | 1018 | 1618 | 591 | 1530 | 1405 | 1563 |
| ωMCA | 2818 | 1242 | 3098 | 4931 | 3914 | 3353 | 2381 | 3380 | 1578 | 6457 | 3582 | 3297 | 3948 | 2748 | 2813 |
| total | 64321 | 43998 | 60685 | 82257 | 71809 | 64323 | 66313 | 73089 | 53446 | 105630 | 68528 | 49696 | 102171 | 69989 | 76378 |

HC for healthy control, PCD for post-cholecystectomy diarrhea, NP for NonPCD, n=5 for each group.

**Table S5**: The ratio of primary bile acids to secondary bile acids in feces of these grouped mice.

| Sample Name | HC1 | HC2 | HC3 | HC4 | HC5 | NP1 | NP2 | NP3 | NP4 | NP5 | PCD1 | PCD2 | PCD 3 | PCD4 | PCD5 |
| --- | --- | --- | --- | --- | --- | --- | --- | --- | --- | --- | --- | --- | --- | --- | --- |
| CA | 4761 | 1838 | 5109 | 8683 | 5170 | 3845 | 2825 | 5580 | 2845 | 10073 | 5120 | 3650 | 4221 | 4672 | 3321 |
| GCA | 22 | 8 | 19 | 37 | 29 | 10 | 12 | 51 | 20 | 28 | 32 | 14 | 14 | 18 | 13 |
| TCA | 1550 | 787 | 1224 | 978 | 1067 | 702 | 1622 | 1043 | 969 | 1255 | 1468 | 1671 | 1080 | 1232 | 1213 |
| UCA | 5838 | 1937 | 5126 | 10134 | 5119 | 3744 | 2744 | 7542 | 3224 | 11443 | 5056 | 3670 | 4033 | 6215 | 3342 |
| ACA | 444 | 337 | 555 | 246 | 329 | 55 | 99 | 146 | 551 | 431 | 220 | 1211 | 800 | 72 | 464 |
| NorCA | 188 | 105 | 267 | 158 | 164 | 142 | 68 | 232 | 254 | 247 | 152 | 197 | 235 | 223 | 207 |
| CDCA | 605 | 649 | 195 | 591 | 536 | 478 | 615 | 541 | 470 | 490 | 539 | 391 | 862 | 890 | 793 |
| TCDCA | 311 | 148 | 219 | 134 | 221 | 99 | 221 | 164 | 168 | 180 | 191 | 285 | 182 | 238 | 176 |
| GCDCA | 8 | 5 | 8 | 8 | 7 | 4 | 4 | 5 | 7 | 6 | 6 | 7 | 8 | 6 | 6 |
| αMCA | 1789 | 1233 | 1282 | 542 | 1032 | 999 | 1128 | 750 | 1217 | 1018 | 1618 | 591 | 1530 | 1405 | 1563 |
| βMCA | 3252 | 1528 | 8390 | 9823 | 9747 | 5337 | 6095 | 3423 | 2810 | 6389 | 8623 | 4056 | 7701 | 4048 | 8300 |
| TβMCA | 1585 | 788 | 1179 | 946 | 1124 | 646 | 1262 | 752 | 733 | 1030 | 1311 | 1774 | 971 | 943 | 919 |
| total primary BAs | 20355 | 9361 | 23573 | 32280 | 24543 | 16062 | 16694 | 20229 | 13268 | 32591 | 24336 | 17515 | 21636 | 19961 | 20318 |
|  |  |  |  |  |  |  |  |  |  |  |  |  |  |  |  |
| DCA | 3321 | 2654 | 4277 | 4689 | 4140 | 4961 | 4201 | 4427 | 2652 | 6294 | 3838 | 4372 | 8219 | 5773 | 5259 |
| TDCA | 206 | 105 | 163 | 87 | 138 | 70 | 171 | 108 | 115 | 121 | 109 | 187 | 198 | 164 | 117 |
| isoDCA | 10 | 1 | 4 | 8 | 9 | 10 | 2 | 5 | 12 | 24 | 3 | 2 | 1 | 1 | 6 |
| GDCA | 9 | 6 | 9 | 10 | 6 | 3 | 4 | 8 | 6 | 6 | 7 | 7 | 7 | 5 | 5 |
| NorDCA | 46 | 29 | 7 | 28 | 13 | 11 | 18 | 17 | 22 | 46 | 6 | 3 | 31 | 4 | 12 |
| 7-ketoDCA | 2 | 41 | 22 | 7 | 42 | 11 | 4 | 10 | 7 | 11 | 4 | 2 | 1 | 4 | 7 |
| LCA | 496 | 484 | 562 | 300 | 363 | 440 | 474 | 350 | 472 | 346 | 288 | 253 | 558 | 623 | 714 |
| TLCA | 92 | 30 | 25 | 14 | 11 | 16 | 21 | 24 | 29 | 22 | 5 | 24 | 32 | 26 | 20 |
| GLCA | 9 | 6 | 13 | 4 | 3 | 1 | 1 | 3 | 1 | 0 | 1 | 14 | 3 | 2 | 1 |
| alloLCA | 4095 | 2624 | 4130 | 2986 | 3025 | 3928 | 4993 | 3922 | 3233 | 2554 | 2765 | 2481 | 6368 | 5095 | 6261 |
| isoLCA | 3679 | 2004 | 3993 | 2760 | 3020 | 3738 | 4553 | 3589 | 2844 | 2594 | 2605 | 2361 | 6218 | 4243 | 5379 |
| 6-ketoLCA | 2533 | 2311 | 1526 | 1544 | 1930 | 632 | 1022 | 914 | 1363 | 2272 | 1574 | 1256 | 1011 | 937 | 1359 |
| 12-ketoLCA | 0 | 1 | 0 | 0 | 1 | 0 | 0 | 0 | 0 | 0 | 0 | 2 | 4 | 0 | 0 |
| 7,12-diketoLCA | 3 | 2 | 5 | 3 | 6 | 2 | 1 | 6 | 2 | 3 | 7 | 5 | 10 | 10 | 1 |
| dehydroLCA | 1584 | 1074 | 1520 | 954 | 1298 | 622 | 892 | 758 | 1360 | 1059 | 1271 | 992 | 1018 | 730 | 1249 |
| MDCA | 6376 | 5308 | 3655 | 2983 | 4122 | 3763 | 4617 | 5482 | 5060 | 5376 | 4389 | 2386 | 6773 | 7360 | 5883 |
| ωMCA | 2818 | 1242 | 3098 | 4931 | 3914 | 3353 | 2381 | 3380 | 1578 | 6457 | 3582 | 3297 | 3948 | 2748 | 2813 |
| TUDCA | 518 | 285 | 814 | 380 | 192 | 443 | 350 | 315 | 190 | 804 | 170 | 467 | 600 | 202 | 294 |
| UDCA | 2 | 4 | 5 | 3 | 6 | 3 | 4 | 1 | 1 | 16 | 1 | 3 | 7 | 3 | 12 |
| βUDCA | 5032 | 4576 | 3592 | 3688 | 5180 | 3616 | 4214 | 5157 | 3015 | 5345 | 5487 | 3164 | 6331 | 4354 | 5445 |
| GUDCA | 5 | 2 | 0 | 3 | 3 | 2 | 1 | 2 | 2 | 3 | 3 | 2 | 4 | 3 | 2 |
| HDCA | 7406 | 5256 | 5399 | 3350 | 4848 | 4310 | 5290 | 6407 | 5315 | 6229 | 5527 | 2477 | 9648 | 8118 | 8950 |
| GHDCA | 20 | 15 | 23 | 11 | 23 | 6 | 4 | 38 | 17 | 7 | 16 | 8 | 7 | 11 | 16 |
| THDCA | 345 | 235 | 76 | 22 | 39 | 22 | 14 | 17 | 67 | 41 | 15 | 10 | 32 | 83 | 20 |
| βHDCA | 2 | 21 | 16 | 19 | 59 | 56 | 22 | 20 | 29 | 38 | 39 | 149 | 29 | 33 | 20 |
| THCA | 1407 | 774 | 971 | 905 | 1056 | 643 | 1574 | 931 | 1000 | 1146 | 1214 | 1628 | 980 | 1036 | 1043 |
| secondary BAs | 40018 | 29087 | 33904 | 29692 | 33447 | 30661 | 34828 | 35891 | 28391 | 40813 | 32926 | 25553 | 52037 | 41570 | 44889 |
|  |  |  |  |  |  |  |  |  |  |  |  |  |  |  |  |
| secondary BAs/primary BAs | 1.966 | 3.107 | 1.438 | 0.920 | 1.363 | 1.909 | 2.086 | 1.774 | 2.140 | 1.252 | 1.353 | 1.459 | 2.405 | 2.083 | 2.209 |

HC for healthy control, PCD for post-cholecystectomy diarrhea, NP for NonPCD, n=5 for each group.
